# MCRS1 modulates the heterogeneity of microtubule minus-end morphologies in mitotic spindles

**DOI:** 10.1091/mbc.E22-08-0306-T

**Published:** 2022-12-15

**Authors:** Alejandra Laguillo-Diego, Robert Kiewisz, Carlos Martí-Gómez, Daniel Baum, Thomas Müller-Reichert, Isabelle Vernos

**Affiliations:** a Centre for Genomic Regulation, The Barcelona Institute of Science and Technology, Barcelona 08003, Spain; b Experimental Center, Faculty of Medicine Carl Gustav Carus, Technische Universität Dresden, 01307 Dresden, Germany; c Simons Center for Quantitative Biology, Cold Spring Harbor Laboratory, Cold Spring Harbor, NY 11724; d Department of Visual and Data-Centric Computing, Zuse Institute Berlin, 14195 Berlin, Germany; e Universitat Pompeu Fabra, Barcelona 08003, Spain; f ICREA, Barcelona 08010, Spain; Indiana University

## Abstract

Faithful chromosome segregation requires the assembly of a bipolar spindle, consisting of two antiparallel microtubule (MT) arrays having most of their minus ends focused at the spindle poles and their plus ends overlapping in the spindle midzone. Spindle assembly, chromosome alignment, and segregation require highly dynamic MTs. The plus ends of MTs have been extensively investigated but their minus-end structure remains poorly characterized. Here, we used large-scale electron tomography to study the morphology of the MT minus ends in three dimensionally reconstructed metaphase spindles in HeLa cells. In contrast to the homogeneous open morphology of the MT plus ends at the kinetochores, we found that MT minus ends are heterogeneous, showing either open or closed morphologies. Silencing the minus end–specific stabilizer, MCRS1 increased the proportion of open MT minus ends. Altogether, these data suggest a correlation between the morphology and the dynamic state of the MT ends. Taking this heterogeneity of the MT minus-end morphologies into account, our work indicates an unsynchronized behavior of MTs at the spindle poles, thus laying the groundwork for further studies on the complexity of MT dynamics regulation.

## INTRODUCTION

The mitotic spindle is a highly complex molecular machinery that assembles during cell division to segregate chromosomes to the daughter cells. It is structurally and functionally defined by its main component, the microtubules (MTs). During mitosis, MTs are nucleated through different pathways, involving the centrosomes, the chromosomes, and preexisting MTs (Meunier and Vernos, 2012; Teixido-Travesa *et al.*, 2012; Lüders, 2016). Altogether, the bipolar spindle consists of MTs with their plus ends at the spindle equator and the minus ends associated with the two spindle poles (i.e., the centrosomes) or with other MT lattices (Kiewisz *et al.*, 2022). Within the spindle of mammalian cells, kinetochore MTs (KMTs) organize into bundles, so-called kinetochore-fibers (k-fibers) that connect chromosomes to the two spindle poles. In addition, astral MTs (AMTs) and interpolar MTs (IMTs) (both termed non-KMTs here) constitute the main body of the spindle, providing additional forces for spindle positioning and chromosome movements by interaction with molecular motors (Meunier and Vernos, 2012, 2016).

The regulation of MT dynamics is fundamental for the overall organization of the spindle and the segregation of the chromosomes. Dynamics can be observed at both MT ends that can stochastically switch between phases of growth and shrinkage (Mitchison and Kirschner, 1984; Kirschner and Mitchison, 1986). MT plus-end dynamics promotes the attachment of MTs to the kinetochores and is important for the assembly of the k-fibers (Mimori-Kiyosue and Tsukita, 2003). The dynamic nature of these attachments is key for error correction, the activity of the spindle assembly checkpoint (SAC), and chromosome segregation (Maiato *et al.*, 2004; Foley and Kapoor, 2013; Lampson and Grishchuk, 2017; Monda and Cheeseman, 2018). Although MT minus-end dynamics seems to play a role in spindle assembly and chromosome movements, little information is currently available on the structure and dynamics of the MT minus ends in the mammalian spindle, as most of them are focused at the spindle poles, an extremely crowded region difficult to observe and manipulate (Akhmanova and Steinmetz, 2019).

In mammalian cells, most MTs are nucleated by the γ-TuRC, a dedicated multiprotein complex. Although MTs in mitosis are nucleated through various pathways, spindle pole–located γ-TuRC plays a crucial role in MT nucleation. In general, MTs nucleated from γ-TuRC are “capped” at their minus ends, and they are therefore stabilized (Consolati *et al.*, 2020; Wieczorek *et al.*, 2020; Zimmermann *et al.*, 2020). However, various studies indicate that MT minus ends depolymerize at the spindle poles (Jiang *et al.*, 2017). Indeed, a movement of tubulin subunits from the spindle equator to the spindle poles was observed by live-cell imaging upon photoactivation of a tubulin stripe in close proximity to the aligned chromosomes in the metaphase spindle (Mitchison, 1989). This phenomenon, named spindle flux, was proposed to play a role in the control of spindle length and in the chromosome segregation (Ganem *et al.*, 2005; Ganem and Compton, 2006; Steblyanko *et al.*, 2020). The current view is that the spindle flux is the result of a combination of MT transport toward the spindle poles and net incorporation of tubulin at the MT plus ends. This incorporation is compensated by the removal of tubulin dimers at MT minus ends at the spindle poles (Kirschner and Mitchison, 1986; Mitchison, 1989; Waters *et al.*, 1996) caused by kinesin-13 depolymerases (Ems-McClung and Walczak, 2010). Consistently, silencing the spindle pole–localized kinesin-13 depolymerase, kif2a, leads to the formation of monopolar spindles, indicating that the regulation of MT minus-end dynamics is essential for bipolar spindle assembly (Ganem and Compton, 2004). Additional support for this idea was also provided by the identification of Microspherule Protein 1 (MCRS1), a RanGTP-regulated protein that localizes to the KMT minus ends and regulates their depolymerization rate at the spindle poles. Spindles assembled in MCRS1-silenced cells have a faster poleward flux, show hyperstretched kinetochores, and form unstable spindles (Meunier and Vernos, 2011).

Several studies indicate that the dynamic state of MTs is associated with specific end morphologies. Cryo-electron microscopy studies of in vitro–assembled MTs showed that fast-growing MTs have flared ends with curved sheet-like protofilaments at their tips, while slowly growing MTs have blunt ends (Simon and Salmon, 1990; Mandelkow *et al.*, 1991; Chretien *et al.*, 1995; Muller-Reichert *et al.*, 1998; Rice, 2018). In contrast, depolymerizing MT ends display outward-curled (also called ramshorn-like) protofilaments (Simon and Salmon, 1990; Mandelkow *et al.*, 1991; Chretien *et al.*, 1995; Muller-Reichert *et al.*, 1998; Rice, 2018)*.* Importantly, closed MT ends have also been observed. MTs nucleated by the γ-TuRC complex in vitro show an electron-dense material, thus “closing” their minus ends (Zheng *et al.*, 1995).

Although MT dynamics in cells is more complex than in vitro due to the presence of a large variety of MT-associated proteins (MAPs), including some that specifically bind to the MT ends, the morphologies of both growing and depolymerizing ends were found to be very similar to those observed in vitro (VandenBeldt *et al.*, 2006). Moreover, recent electron microscopy studies of plastic-embedded spindles in *Caenorhabditis elegans* also revealed flared and curled MT end morphologies (O’Toole *et al.*, 2003), suggesting that the spindle MTs may be in different phases of polymerization and depolymerization, respectively. MTs having a blunt end may be pausing, polymerizing, or depolymerizing, and therefore it is difficult to categorize them in any specific dynamic state. However, more recent tomographic reconstructions in vitro and in cells display similar bent MT tips in growing and shortening MTs, indicating that dynamic states cannot be distinguished that easily (McIntosh *et al.*, 2018; Gudimchuk *et al.*, 2020; Gudimchuk and McIntosh, 2021).

Interestingly, partial reconstructions of metaphase spindles in U2OS cells displayed a mixture of closed and open morphologies at the pole-facing MT ends (i.e., at the putative minus ends; Kamasaki *et al.*, 2013). Consistently, studies performed in early *C. elegans* embryos showed that the minus ends of KMTs have heterogeneous morphologies described as either closed or open (O’Toole *et al.*, 2003). The presence of a “cap-like” structure at the MT minus ends associated with either spindle pole bodies in *Schizosaccharomyces pombe* or mitotic centrosomes in *C. elegans* embryos was interpreted as the γ-TuRC (O’Toole *et al.*, 2003; Hoog *et al.*, 2011; Teixido-Travesa *et al.*, 2012). However, a systematic characterization of MT minus ends in mitotic spindle poles of mammalian cells is currently not available.

Here, we set out to gain novel insights into the MT minus-end morphologies in the metaphase spindle of human cells using electron tomography. We found that MT minus ends can have either closed or open morphologies in a proportion that is modified upon silencing by the MT minus-end regulator MCRS1. The observed heterogeneity of KMT minus-end morphologies suggests a complex mechanism of regulation of their dynamics.

## RESULTS

### Different MT minus-end morphologies coexist at the spindle poles

**FIGURE 1:**
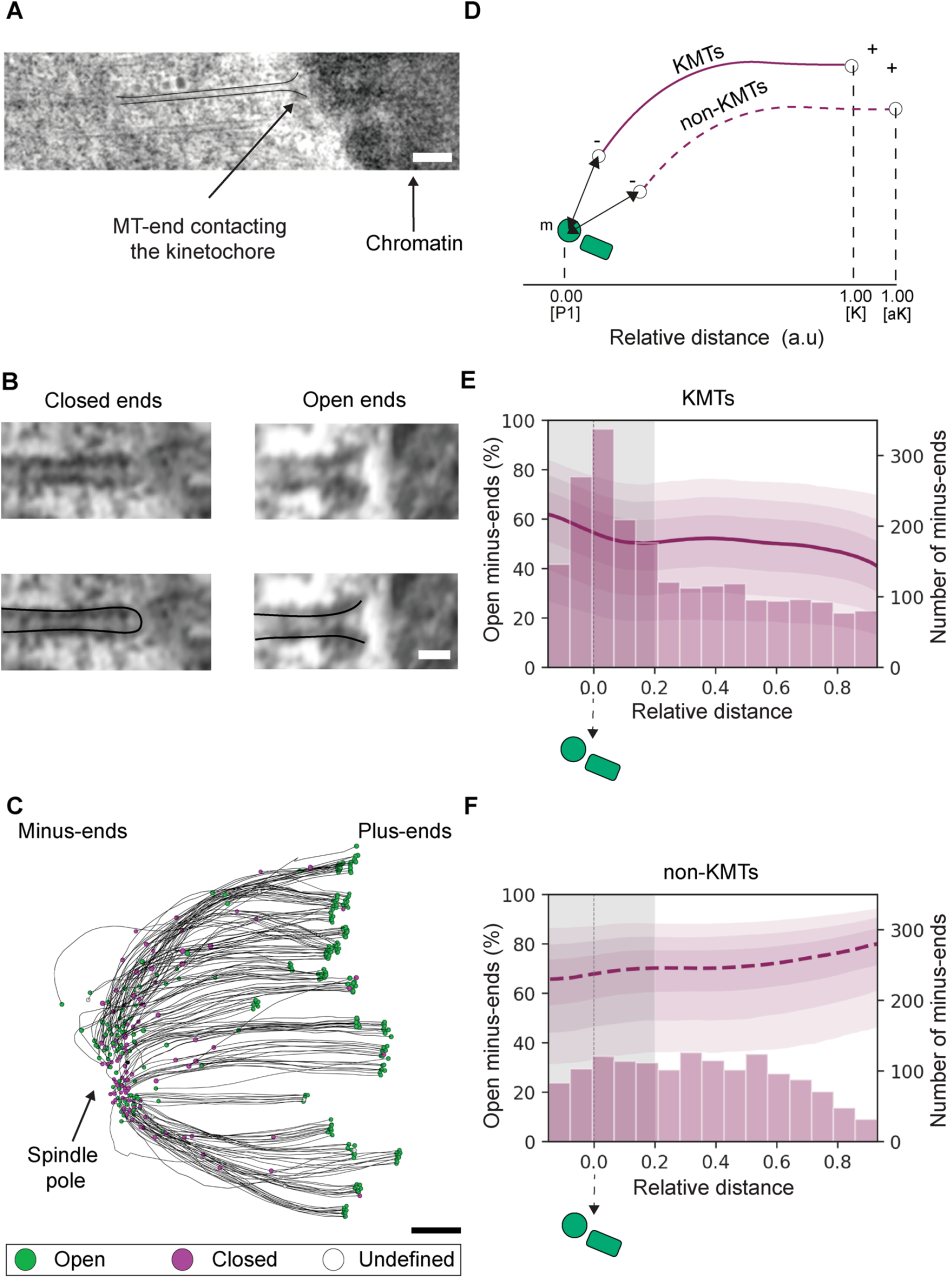
Quantitative analysis of MT minus-end morphologies. (A) Tomographic slice showing association with a chromosome (arrow) as visualized in a HeLa cell at metaphase. In this study, such an MT is defined as a KMT (top view, tomographic slice with overlay). Scale bar, 100 nm. (B) Visualization of a closed and an open MT end morphology (top row, tomographic slice; bottom row, tomographic slices with overlays for better illustration). MT ends showing an electron-dense cap are classified as closed (left panels). Curved or sheet-like ends are classified as open (right panels). Scale bar, 25 nm. (C) Orthogonal projection (top view) of a 3D model showing a quarter volume of a control spindle (Supplemental Table S2). KMTs are depicted as black lines, and their end morphologies are indicated as open (green circles), closed (purple circles), or undefined (open circles). The position of the spindle pole is indicated by an arrow. Scale bar, 500 nm. (D) Schematic drawing illustrating the positional analysis of both KMT and non-KMT minus ends. The position of each MT minus end along the spindle pole axis is given as a relative distance. The relative distance of each KMT minus end along the half-spindle axis is defined by the position between the pole [P1, 0] and the kinetochore [K, 1]. The relative position of non-KMT minus ends is defined by the position of the pole and the average position of all kinetochores in the spindle [aK, 1]. The absolute distance (arrows) is determined by measuring the 3D distance of each MT minus end to the nearest mother centriole (m). A KMT is indicated by a solid purple line, a non-KMT by a dashed purple line. (E) Histogram showing the absolute number of KMT minus ends (right axis) at different relative distances. The graph also shows the estimated percentage of open minus ends at each given relative position (left axis). The different levels of shadow around the line (purple) correspond to the CIs, representing 95, 80, and 50% probabilities to observe the true value within these intervals. The shaded area (gray) represents the MT spindle pole association area. This area was defined as the region of the spindle pole with the highest concentration of KMT minus ends as observed in the histogram. The total number of classified KMT minus ends for control spindles was *n* = 2071. (F) Histogram showing the absolute number of non-KMT minus ends in the MT spindle pole association area. The total number of classified non-KMT minus ends is *n* = 1516.

We used three-dimensional (3D) reconstructions of whole mitotic spindles assembled in HeLa cells (Kiewisz *et al.*, 2022) to analyze the morphology of the MT ends (Figure 1A, Supplemental Figure S1, A, C, E, and G, and Supplemental Video 1; Supplemental Tables S1 and S2). In these control cells (called “control” hereafter), we defined two main classes of ends: closed ends that have an electron-dense “cap” (Figure 1B, left panels; Supplemental Figure S5A) and open ends that typically have a flared morphology (Figure 1B, right panels; Supplemental Figure S5B). Other types of MT ends were classified as “undefined.”

**Figure:** Movie S1 **Generation of a 3D model assembled by joining serial electron tomograms** Series of tomograms and corresponding 3D model of a control spindle #3. The video illustrates the stacking of serial tomograms to increase the volume of reconstruction. KMTs are shown as red lines. This video corresponds to the spindle shown in **Fig. 1C**.

We first focused on the MTs forming the k-fibers (i.e., the KMTs), strictly defined as those directly associated through their plus ends to the outer kinetochores at the chromosomes (Kiewisz *et al.*, 2022). In agreement with the dynamic nature of the KMT plus ends (Cheeseman and Desai, 2008), close examination of the KMT end morphology at the kinetochore showed that they were open (green circles) and flared (Figure 1, B, right panel, and C) (O’Toole *et al.*, 2003). We then focused on the opposite end of the KMTs away from the kinetochore, defining this as the minus end. KMT minus ends had either closed (Figure 1C, circles in purple) or open morphologies (Figure 1C, circles in green). In the context of previous work on MT minus-end dynamics and morphology (Hoog *et al.*, 2011), this suggested to us that the heterogeneous morphologies of the KMT minus ends reflect the coexistence of different dynamic states.

Previous work had indicated that about half of the KMT minus ends are located at the spindle poles (defined as the MT–centrosome interaction area), while the other half is distributed along the half-spindle (Kiewisz *et al.*, 2022). Extending this previous analysis, we were also interested to know whether there was a correlation between the KMT minus-end morphologies and the positioning in the spindle. For this, we plotted the distribution of KMT minus-end relative distance on the kinetochore-to-spindle axis (Figure 1D). The kinetochore-to-spindle axis was defined as the distance between the closest spindle pole (position = 0) and the individual kinetochores (position = 1). Additionally, we also defined the MT–centrosome interaction area as twice the half-width. We then determined the proportion of open minus ends at relative distances along the kinetochore-to-spindle axis (Figure 1E). The inferred proportion of the open minus ends in the proximity of the centriole pair (defining the spindle pole) was 52.97% (95% credible interval [CI] = [21.48, 77.43]%) (Figure 1E, gray vertical area *D* < 0.2, region where the majority of KMT minus ends are located; Supplemental Table S3). Therefore, about half of the KMT minus ends at the spindle poles were open, suggesting that they are in different dynamic states.

We then asked whether KMTs belonging to the same k-fiber might have similar minus-end morphologies indicative of a synchronized dynamic state. To address this question, we performed a model comparison using a likelihood ratio test between models that included or ignored the k-fiber as a random effect. We found no evidence of clustering of open minus ends at specific k-fibers (*p* value = 1). This suggested that the minus-end morphology of any given KMT within a k-fiber is independent of the minus-end morphologies of the other KMTs in the same k-fiber.

To determine whether the mixture of minus-end morphologies suggestive of mixed dynamic states is specific for the KMTs or a more general feature of the spindle MTs, we extended our studies to the non-KMTs. For these MTs, we defined the average position of all kinetochores (Figure 1D, aK) as position 1 on the half-spindle axis. In addition, the polarity of the non-KMTs was determined by the positioning of the ends along the half-spindle axis. The end of each non-KMT closer to the spindle pole was defined as the minus end, with the other end assigned as the plus end. The end morphologies of non-KMTs were also classified as either closed or open. We then estimated the proportion of open minus ends at each relative distance. We found that 64.04% (CI = [33.00, 86.22]%) of the non-KMT minus ends were open in the region near the centriole pair (Figure 1F, gray area; Supplemental Table S3). Again, these data showed that MT minus ends have heterogeneous morphologies, overall suggesting the coexistence of different dynamic states.

### MCRS1 silencing induces changes in k-fiber ultrastructure and spindle shape

**FIGURE 2:**
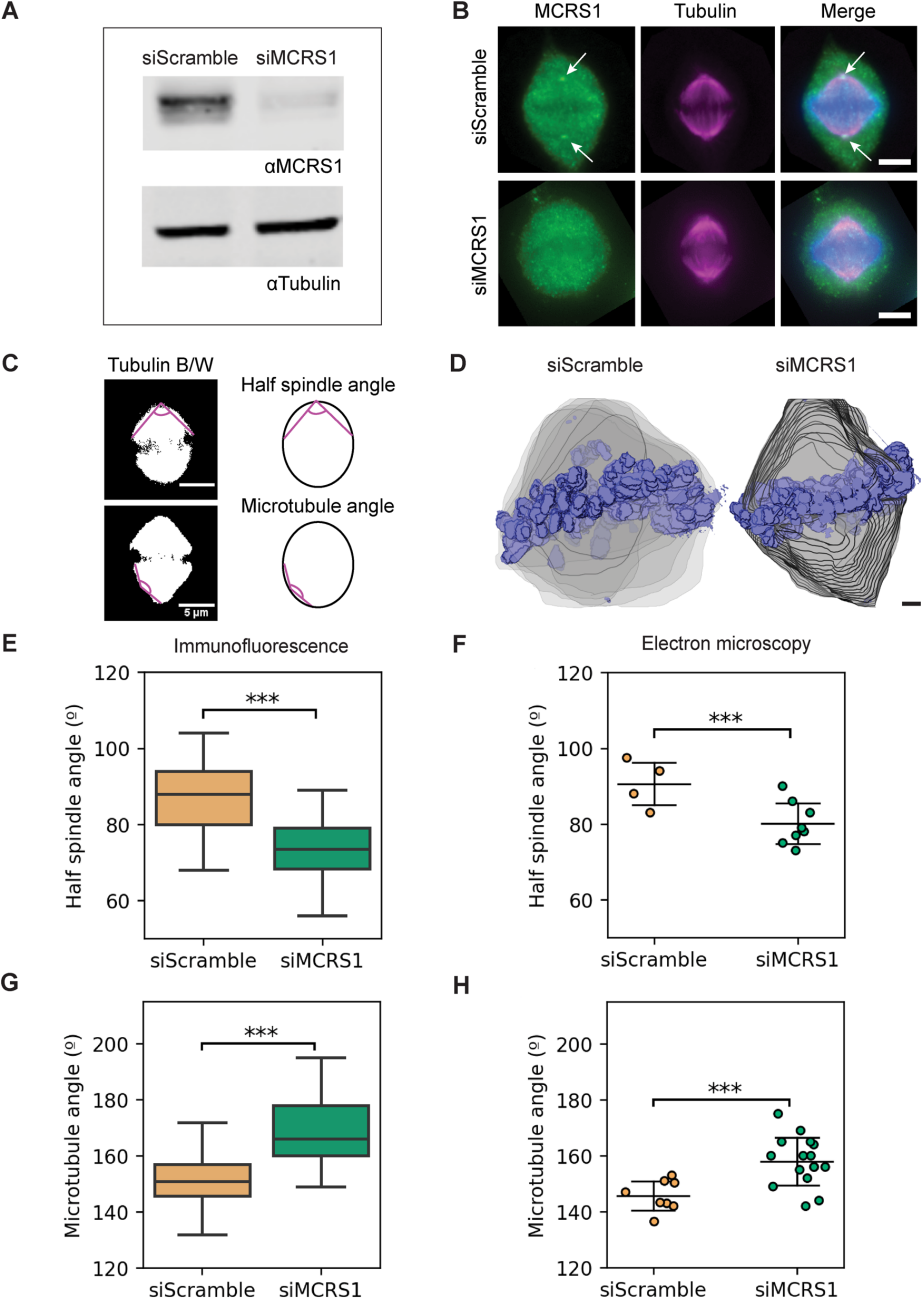
Characterization of metaphase spindles in MCRS1-silenced cells. (A) Western blot analysis showing the levels of MCRS1 in control and MCRS1-silenced cells. Tubulin levels were used as a loading control. (B) Immunofluorescence staining of siScramble (top panel) and MCRS1-silenced cells (bottom panel). MCRS1 is shown in green, DNA in blue, and tubulin in magenta. MCRS1 staining can be observed as tiny spots at spindle poles in the control cell (arrows). Upon silencing, MCRS1 cannot be detected anymore. Scale bars, 5 μm. (C) Black/white images of control spindle (top, left) and a silenced spindle (bottom, left). The magenta lines show the angles that were measured for the half-spindle (top, right) and for the MTs in the outward position of the spindle (bottom, right). Scale bars, 5 μm. (D) Three-dimensional models of spindle shape in siScramble and siMCRS1 cells were obtained from low-resolution screening by transmission electron microscopy (Supplemental Table S2). The outline of the spindle volume in each serial section is shown in gray and chromosomes in blue. Scale bar, 1 µm. (E) Boxplot showing the quantification of the half-spindle angle obtained from immunofluorescence images. The boxes show the upper and lower quartiles, and the whiskers show the minimum and maximal values excluding outliers; the line inside the box indicates the median. Asterisks show a significant difference according to a linear regression model considering two different experiments with a *p* value = 4.729 × 10^–8^ (siScramble: *n* = 105; siMCRS1: *n* = 110). (F) Scatterplot showing the quantification of the half-spindle angle from electron microscopic images. The line shows the mean, and the error bars represent ± SD. Asterisks show significant differences according to a two-tailed Student’s *t* test with a *p* value = 0.017 (siScramble: *n* = 4; siMCRS1: *n* = 8). (G) Boxplot showing the quantification of the half-spindle angle obtained from immunofluorescence images. Asterisks show a significant difference according to a linear regression model considering two different experiments with a *p* value = 7.486 × 10^–26^ (siScramble: *n* = 201; siMCRS1: *n* = 161). (H) Scatterplot showing the quantification of the MT angle obtained from electron microscopic images (two-tailed Student’s *t* test with a *p* value = 0.002 (siScramble: *n* = 8; siMCRS1: *n* = 16).

MCRS1 was shown to associate with the k-fiber minus ends in metaphase and regulate their dynamics (Meunier and Vernos, 2011). Therefore, we decided to examine the changes in the MT end morphologies in spindles assembled in MCRS1-silenced cells by electron tomography. Because small interfering RNA (siRNA)-based gene silencing may not be homogeneous in a cell population, we first carefully looked for morphological spindle features that could be altered in MCRS1-silenced cells and used as a signature for selecting the spindles to be processed for electron tomography. Western blot analysis of MCRS1-silenced cells showed a reduction in the target protein level of close to 90% (Figure 2, A and B, and Supplemental Figure S3). High-contrast light microscopy images revealed specific changes in the half-spindle shape and the outer spindle MT angle (Figure 2C and Supplemental Figure S4). siScramble cells (i.e., transfected with random siRNA) showed an average half-spindle angle of 80.81° ± 10.60° (Figure 2E, *n* = 105). In contrast, MCRS1-silenced cells had an average half-spindle angle of 71.5° ± 8.05° (*n* = 110; *p* value = 4.729 × 10^–8^), significantly narrower than in siScramble cells. In addition, siScramble cells showed an average angle for the outer MTs of 152.34° ± 9.62° (Figure 2G, *n* = 201), whereas siMCRS1-depleted cells displayed an average angle for the outer MTs of 167.51° ± 10.72° (*n* = 161, *p* value = 7.486 × 10^–26^). Thus, the outer MTs in MCRS1-depleted cells were significantly straighter than in siScramble cells. Similar differences were also clearly observed in 3D models after processing both siScramble and MCRS1-silenced cells for electron tomography. In stacked serial plastic sections (Figure 2D), siScramble cells had a half-spindle angle of 90.62° ± 6.41° (Figure 2F; Supplemental Video 2; *n* = 4), whereas MCRS1-silenced spindles had a significantly lower half-spindle angle of 80.13° ± 5.77° (Supplemental Video 3; *n* = 8, *p* value = 0.017). Moreover, siScramble spindles showed an average outer MT angle of 145.77° ± 5.55° (Figure 2H; Supplemental Video 3; *n* = 8), whereas siMCRS1 spindles displayed a significantly higher average outer MT angle of 158.00° ± 8.74° (*n* = 16, *p* value = 0.002). From these data, we concluded that the depletion of MCRS1 caused a reduction in the half-spindle angle and a simultaneous increase in the MT angle.

**Figure:** Movie S2 **Three-dimensional model of a low-resolution spindle as used for the analysis of spindle geometry in a siScramble cell** Series of electron micrographs of a spindle in a siScramble cell #2. The stacking of serial tomograms to generate a 3D model of the spindle volume is illustrated. The spindle on each section is indicated by a transparent gray area. Chromosomes are shown in blue. This video corresponds to the spindle shown in **Fig. 2D, left panel**.

**Figure:** Movie S3 **Three-dimensional model of a low-resolution spindle as used for the analysis of spindle geometry in a siMCRS1 cell** Series of electron micrographs of a spindle in a siMCRS1 cell #5. The stacking of serial tomograms to generate a 3D model of the spindle volume is illustrated. The spindle on each section is indicated by a transparent gray area. Chromosomes are shown in blue. This video corresponds to the spindle shown in **Fig. 2D, right panel**.

These parameters were then used for the selection of spindles from siScramble cells and MCRS1-silenced cells for 3D reconstruction. To obtain representative data in particular for spindles assembled in MCRS1-silenced cells, we aimed at obtaining data from several different spindles (Supplemental Videos 4 and 5). We then evaluated whether reconstructions of a quarter of the spindle volume would be representative of a full spindle. Using the tomograms from control samples, we divided a full spindle into four symmetrical quarters and measured the number of KMTs per k-fiber and the outer-kinetochore distance (Supplemental Figures S6 and S7). We found no differences between these values and those obtained from the full spindle tomogram analysis. This suggested that the quarter spindles are representative of the corresponding full spindle. Therefore, we decided to reconstruct quarters from two spindles from siScramble cells and five from MCRS1-silenced cells, which allowed us to increase the number of analyzed individual data sets. To determine whether all the selected spindles were at a similar stages in mitosis, we compared the average distances between the outer kinetochores of sister k-fibers for all our data sets. The average interkinetochore distance in the selected spindles was 1.06 µm ± 0.21 µm (*n* = 28) in siScramble cells and 1.06 µm ± 0.21 µm (*n* = 55) in MCRS1-silenced cells. Control spindles showed an average value of 1.07 µm ± 0.21 µm (*n* = 146; siScramble vs. Control, *p* value = 0.108; siScramble vs. siMCRS1, *p* value = 0.240). Additionally, the values measured for Control and siScramble cells were in agreement with previous studies (Kiewisz *et al.*, 2022). The high similarity in the interkinetochore distance in all these conditions indicated that the selected spindles were captured at similar mitotic stages.

**Figure:** Movie S4 **Generation of a 3D model from joined serial electron tomograms displaying spindle siMCRS1 #1** Series of tomograms and corresponding 3D model of spindle siMCRS1 #1. The stacking of serial tomograms to increase the tomographic volume is illustrated. Non-KMTs are shown as yellow lines, and KMTs are illustrated in red. This spindle reconstruction is not shown in any of the presented figures but has been added to show that the morphological alterations upon MCRS1-silencing are consistent between different 3D models. This video corresponds to spindle siMCRS1 #1 as given in **Table 1**.

**FIGURE 3:**
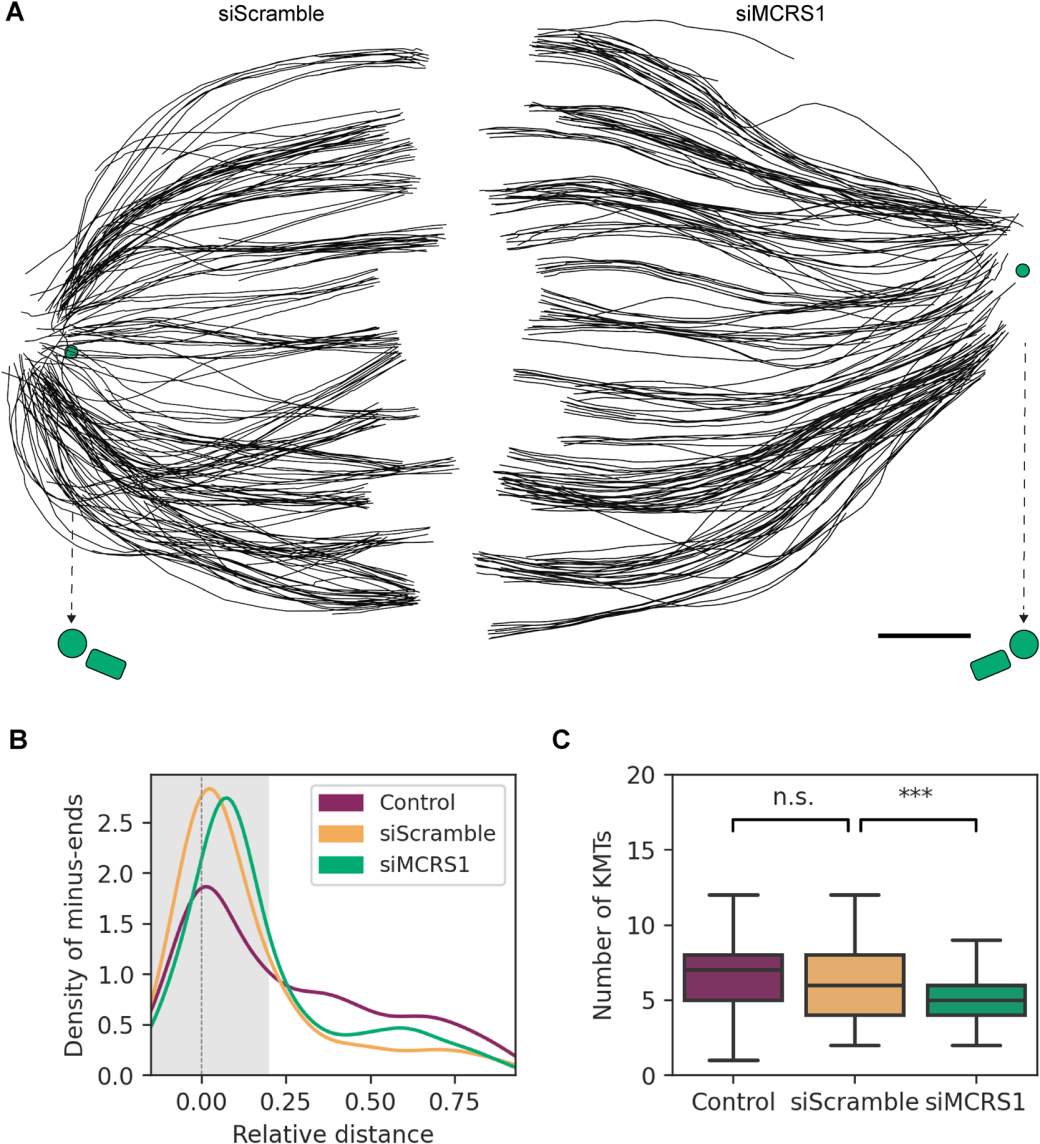
Shape of k-fibers in siScramble and MCRS1-silenced spindles. (A) Orthogonal projections (top views) of 3D reconstructions of quarter spindles in a siScramble cell (left, Scramble spindle #2) and a siMCRS1 cell (right, MCRS1 spindle #5) showing k-fibers as black lines (Supplemental Table S2). The spindle poles are indicated by green circles. The mother centrioles of the spindle poles are indicated by arrows (with dashed lines). Scale bar, 1 µm. (B) Line plot representing the Gaussian kernel density distribution of k-fiber minus ends at different relative distances from the mother centriole (position = 0) to the kinetochores (position = 1). The shaded area indicates the MT centrosome association area. The shaded area represents the MT spindle pole association area. (C) Boxplot representing the number of KMTs per k-fiber in the three conditions. The boxes show the upper and lower quartiles, and the whiskers show the minimum and maximum values excluding outliers; the line inside the box indicates the median. Asterisks show significant differences according to a GLM with a *p* value = 2 × 10^–4^. n.s. stands for nonsignificant with a *p* value = 0.599 (Control: *n* = 226, siScramble: *n* = 44, siMCRS1: *n* = 106).

**Figure:** Movie S5 **Generation of a 3D model from joined serial electron tomograms displaying spindle siMCRS1 #5** Series of tomograms and corresponding 3D model of spindle siMCRS1 #5. The stacking of serial tomograms to increase the tomographic volume is illustrated. KMTs are shown as red lines. This video corresponds to the spindle shown in **Fig. 3A**.

We then analyzed the 3D reconstructions of all spindles. MCRS1-silenced spindles showed distinct ultrastructural features. The k-fibers appeared straighter in siMCRS1 compared with siScramble spindles (Figure 3A; Supplemental Figures S2 and S8; Supplemental Videos 6 and 7; also see Videos 4–6 from Kiewisz *et al.*, 2022). Interestingly, the KMTs did not reach the centriole pair area at the spindle poles in siMCRS1 spindles, whereas the centriole pair was embedded within the mass of KMT minus ends in the siScramble spindles. Consistently, the quantification of MT minus-end distribution revealed that the majority reached the centrioles in control spindles (Figure 3B, mean peak at position = 0.03) and siScramble spindles (Figure 3B, mean peak at position = 0.04, *p* value = 0.201). In contrast, the MT minus ends in siMCRS1 spindles peaked at a mean relative position of 0.07, farther away from the centrioles (Figure 3B, *p* value = 0.018; siScramble vs. siMCRS1 at *D* < 0.2). The displacement of MT minus ends away from the spindle pole could result from a higher MT minus-end depolymerization, which would be consistent with the proposed role of MCRS1 in controlling the rate of MT minus-end depolymerization. We then determined the number of KMTs per k-fiber in the three different conditions (Figure 3C) and evaluated the putative differences using a generalized linear model (GLM) with Poisson likelihood and log link function, using siMCRS1 and siScramble data as covariates. We found that the number of KMTs per k-fiber was reduced in MCRS1-silenced cells, with an average of 7.27 KMT (*n* = 106, *p* value = 0.0002) instead of 9.19 KMTs per k-fiber (*n* = 44) in siScramble spindles. No significant differences were found between this value in siScramble and control cells (8.93 KMTs per k-fiber in control cells, *n* = 226, *p* value = 0.599). Altogether, the morphological alterations in MCRS1-silenced spindles support previously proposed changes in the dynamics of the MT minus ends (Meunier and Vernos, 2011).

**Figure:** Movie S6 **KMTs end morphology in spindle siScramble #2** Three-dimensional model of KMTs with annotated end morphology. KMTs are indicated as black lines. Open KMT ends are labeled with green spheres, closed ends with purple spheres and undefined ends with white spheres. This video corresponds to the spindle shown in **Fig. 3A**

**FIGURE 4:**
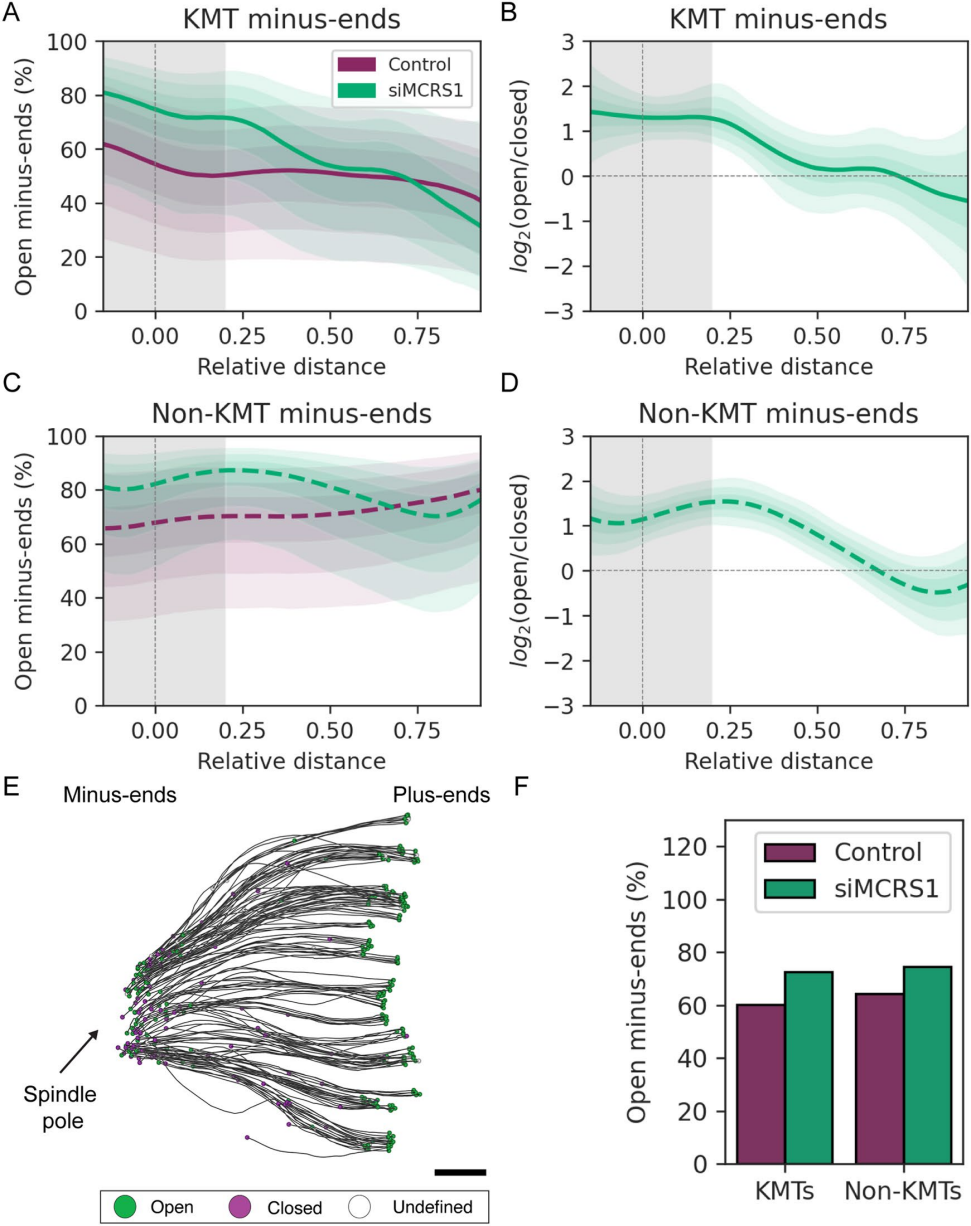
MT minus-end morphology in MCRS1-silenced cells. (A) Estimated percentage of open KMT minus ends plotted against the relative distance on the half-spindle length (position 0 = mother centriole, position 1 = kinetochores) in control cells (purple line; *n* = 1707) and siMCRS1 cells (green line; *n* = 541). The shadow around the lines (purple and green) represents the CI with a 95% probability to observe the true value within this interval. The shaded area (gray) represents the MT spindle pole association area. (B) Posterior distribution of the comparison of the log2(open/closed) of siMCRS1 vs. control (as shown in A) in KMTs (green line). The shadow around the line (green) represents the 95% CI. (C) Estimated percentage of open non-KMT minus ends plotted against the relative distance on the half-spindle length in control cells (purple line, *n* = 1084) and siMCRS1 cells (green line, *n* = 3115). The shadow around the line (green) represents the 95% CI. (D) Posterior distribution of the comparison of the log_2_(open/closed) of siMCRS1 vs. control (as shown in C) in non-KMTs (green line). The shadow around the line (green) represents the 95% CI. (E) Three-dimensional model (top view) showing KMTs in a quarter reconstruction of a siMCRS1 cell (siMCRS1 #5). KMTs are depicted as black lines, and their end morphologies are indicated as open (green circles), closed (purple circles), or undefined (open circles). The position of the spindle pole is indicated by an arrow. Scale bar, 500 nm. (F) Bar representation of the estimated percentage of open KMTs (Control: 60.21%, *n* = 2064; siMCRS1: 72.66%, *n* = 848) and non-KMT minus ends (Control: 64.30%, siMCRS1: 74.51%, *n* = 893; siMCRS1, *n* = 4119) in control cells (purple) and siMCRS1 cells (green) in the spindle pole region (*D* < 0.2).

**Figure:** Movie S7 **KMT end morphology in spindle siMCRS1 #5** Three-dimensional model of KMTs with annotated end morphology. KMTs are indicated as black lines. Open KMT ends are labeled with green spheres, closed ends with purple spheres and undefined ends with white spheres. This video corresponds to the spindle shown in **Fig 3A.** and **Fig. 4F**

### Silencing of MCRS1 increases the proportion of open minus ends of all spindle MTs

To explore more specifically the impact of MCRS1 silencing on the spindle MTs, we then quantified the morphology and relative distribution of their minus ends within the spindle. We selected the KMTs in the reconstructions of the siMCRS1 spindles as described above and manually classified their minus-end morphologies into three categories: open, closed, and undefined (Figure 1B; Supplemental Figure S5). Next, we determined the relative distance of each KMT minus end along the half-spindle axis. The percentage of open KMT minus ends in the siMCRS1 spindles was 75.28% (CI = [44.83, 92.32]%, *n* = 386) in the region surrounding the centriole pair (*D* < 0.2; Figure 4A; Supplemental Video 7, green line; Supplemental Table S3), whereas it was 56.17% in control cells (CI = [25.05, 79.23]%, *n* = 2071). To compare the two percentages, as percentages are bound between 0 and 100, we used the log2 transformation of the ratio open/closed. In this new scale, 0 means that there are the same numbers of open and closed minus ends; log2(open/closed) = 1 indicates that there are two times more open than closed minus ends and –1 that there are two times more closed than open minus ends. This quantity is unbound and symmetric regardless of the group order, so it is more appropriate to represent differences between two groups in percentages. The log2(open/closed) was 1.38 higher in siMCRS1 cells compared with control cells around the centrioles (CI = [0.31, 2.39]; Figure 4B). Our data show that MCRS1 reduction leads to an increase in the percentage of “uncapped” KMT minus ends.

We then examined the minus-end morphologies of non-KMTs at the spindle poles following the procedure described above. Owing to the high number of this class of MTs, we performed this analysis on an unbiased randomly selected number of non-KMTs. We found that the estimated percentages of open non-KMT minus ends in the region near the centriole pair (*D* < 0.2) were 80.76% (CI = [53.16, 93.31]%) in MCRS1-silenced cells and 64.04% (CI = [33.00, 86.21]%) in control cells (Figure 4C and Supplemental Table S3). As before, we used the log2 transformation of the ratio open/closed to compare the two percentages. The log2(open/closed) was 1.35 higher in siMCRS1 cells compared with control cells for non-KMTs (CI = [0.56, 2.14], Figure 4D) for the non-KMT minus ends. This analysis revealed that the non-KMT minus-end morphologies are also altered close to the spindle pole in the absence of MCRS1.

Previous experiments suggested that MCRS1 function was specific for the regulation of the KMT minus-end dynamics (Meunier and Vernos, 2011). To directly address this, we analyzed our data to determine whether the changes in the ratios of close and open minus ends for KMTs and non-KMTs in control versus siMCRS1 cells were similar or not. We eliminated the distance dependence of our analysis and restricted our quantification to the spindle-pole region (*D* < 0.2) for both MT populations (Figure 4F). Considering both categories together, the change in log(open/closed) between siMCRS1 and control cells for non-KMTs was not significantly different from that in KMTs (Δlog(open/closed) = –0.07, *p* value = 0.79). Altogether, these results suggested that MCRS1 silencing has similar impacts on the morphology of both KMT and non-KMT minus ends at the spindle poles with an increase in the percentage of open ends in the absence of MCRS1.

## DISCUSSION

The depolymerization of MT minus ends has been postulated to play an essential role in the control of spindle size, k-fiber dynamics, and chromosome movements (Waters *et al.*, 1996; Barisic *et al.*, 2021). However, there is currently little information on the precise mechanism that establishes and controls MT minus-end dynamics at the spindle poles. An analysis of MT ends at spindle poles is hampered by the fact that the centrosomes are extremely dense locations in mitotic spindles, thus making it impossible to apply light microscopy for analysis of MT dynamics at the level of individual polymers. Direct visualization of MT ends by electron microscopy, however, can provide insightful clues about the end morphology of individual MTs (O’Toole *et al.*, 2003; Redemann *et al.*, 2017). As an example, the majority of KMT plus ends are dynamic and show mainly open and flared morphologies (VandenBeldt *et al.*, 2006; McIntosh *et al.*, 2008, 2013, 2018), suggesting that there is indeed a correlation between MT end morphologies and dynamic states (Hoog *et al.*, 2011). In this context, we aimed to analyze MT minus-end morphology to infer their dynamics. For this, we applied large-scale electron tomography with single-MT resolution (Redemann *et al.* 2017; Kiewisz *et al.*, 2022) and directly visualized MT minus-end morphologies at mitotic spindle poles in human cells in metaphase.

**FIGURE 5:**
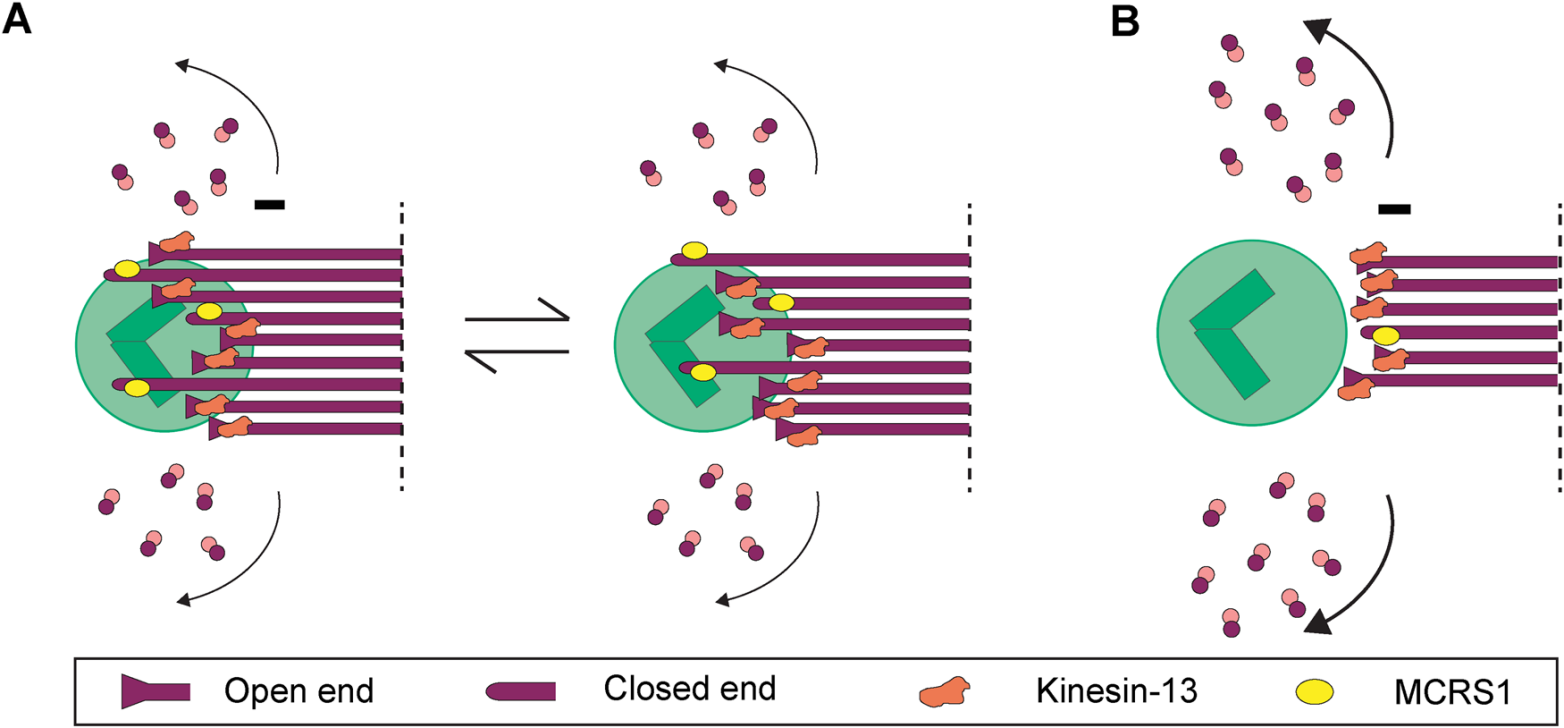
Proposed model of KMT minus-end dynamics regulation in mammalian metaphase. (A) Illustrations of the regulation of KMT minus-end dynamics in the metaphase of control cells. (B) Illustration of minus-end dynamics in response to MCRS1 silencing.

Using this approach, we show that MT minus ends do not have homogeneous morphologies. We further show that both KMTs and non-KMTs display a mixture of both closed and open minus ends, in similar proportions (Figure 5). The closed conformation that we observed for a large proportion of MT minus ends is reminiscent of previous reports on nondynamic MT minus ends capped by the γ-TuRC (Zheng *et al.*, 1995). This suggests that the closed-end morphology may correspond to stable and/or anchored MTs. The mixture of morphologies that we observed for the MT minus ends in the spindle may suggest that their depolymerization at the spindle pole is not synchronous, not even for KMTs within the same fiber.

We observed a significant increase in the proportion of open minus ends at the spindle poles upon silencing of the proposed regulator of MT minus-end dynamics, MCRS1 (Meunier and Vernos, 2011). In addition, 3D reconstruction of spindles revealed an increase in the relative distance of the KMT minus ends to the centriole pair in these cells (Figure 4F), which might be consistent with an increase in MT minus-end depolymerization (Meunier and Vernos, 2011). Moreover, the loss of curvature of the KMTs (Figure 3A and Supplemental Figure S7) and the characteristic sigmoidal shape (recognized in all siMCRS1 spindles) suggest an increase in tension within the spindle, consistent with previous reports about the forces exerted by MT minus-end depolymerization on the attached chromosomes (Waters *et al.*, 1996; DeLuca *et al.*, 2006).

MCRS1 was identified as a novel RanGTP-regulated factor that associates specifically with the k-fiber minus ends in cold-treated cells (Meunier and Vernos, 2011). We found here that both KMT and non-KMT minus-end morphologies change in MCRS1-silenced cells, suggesting that MCRS1 may in fact associate with both MT populations in the spindle. However, because it was recently reported that KMTs directly interact with many non-KMTs near the spindle pole (Kiewisz *et al.*, 2022), it is possible that some of the MTs we classified as being non-KMT are in fact part of the k-fibers. Alternatively, MCRS1 may have a more general function in regulating MT minus-end dynamics within the spindle than previously proposed.

It is interesting to speculate about the nature of the complex that associates with the MT minus ends to generate a cap structure. The γ-TuRC with an approximate molecular weight of 2353.97 kDa appears as a cone-like structure as observed in vitro (Keating and Borisy, 2000; Moritz *et al.*, 2000; Wiese and Zheng, 2000; Kollman *et al.*, 2015; Consolati *et al.*, 2020; Drutovic *et al.*, 2020; Wieczorek *et al.*, 2020; Zimmermann *et al.*, 2020; Brilot *et al.*, 2021). The minus ends of some MTs may be capped by this complex, and one would not expect these MTs to be dynamic. Instead, because MT minus ends are thought to depolymerize at the spindle pole, it is tempting to speculate that the electron-dense material that generates the close morphology could correspond to other MT minus-end binding complexes that could have more dynamic modes of binding, such as the MCRS1-KANSL-complex (Meunier *et al.*, 2015), ASPM-katanin (Jiang *et al.*, 2017), and/or NuMA/dynein (Elting *et al.*, 2014) (Figure 5).

Recent data suggested that MT minus-end depolymerization and spindle flux can be uncoupled (Steblyanko *et al.*, 2020). Instead, our data suggest that there is a direct correlation between the proportion of open MT minus ends and the speed of the spindle flux because it was reported to increase in the absence of MCRS1 (Meunier and Vernos, 2011). The question remains, however, of how cells can undergo constant spindle flux when MT minus ends are not synchronously depolymerizing. Unfortunately, our data can provide only a snapshot of the MT minus-end morphologies in spindles at a given time point. Most likely, the binding/unbinding of components at the MT minus ends is stochastic. In such a model, MT minus ends would overall depolymerize, although individual MTs may pause, which would be visible as a heterogeneity in the morphology of individual ends (Figure 5). A similar situation but with a net polymerization instead of a depolymerization has been described for MT plus ends (VandenBeldt *et al.*, 2006). The putative mechanism underlying MT minus-end dynamics at the spindle poles, therefore, still needs to be addressed in the future (Chen *et al.*, 2019; Dudka *et al.*, 2019).

## MATERIALS AND METHODS

Request a protocol through *Bio-protocol*.

### Standard techniques

#### Cell culture.

HeLa (Kyoto) cells were grown at 37°C in a 5% CO_2_ atmosphere. The cells were cultured in DMEM (Thermo Fisher Scientific, Waltham, MA) containing 4.5 g/l glucose and supplemented with l-glutamine with sodium pyruvate (Lonza, Basel, Switzerland), 10% fetal bovine serum (Thermo Fisher Scientific, Waltham, MA), and an antibiotic cocktail containing 100 μg/ml streptomycin and 100 U/ml penicillin (Sigma-Aldrich, St. Louis, MO). For regular maintenance, HeLa cells were split with 0.25% trypsin (Sigma-Aldrich, St. Louis, MO) when reaching around 80–90% confluency.

#### RNA interference.

Cells were seeded at 150,000 cells/ml in 75 cm^2^ flasks (Sigma-Aldrich, St. Louis, MO) the day before transfection. Per flask, 500 pmol of siRNA and 25 μl of Lipofectamine RNAiMax transfection reagent (Life Technologies, Carslbad, CA) were used and scaled accordingly for smaller volumes. Transfection was performed following the manufacturer’s protocol. After 48 h posttransfection, cells were split and seeded. At 60 h, cells were transfected as described above using siRNA purchased from Dharmacon (Lafayette, CO) as previously described (Meunier and Vernos, 2011) using the following sequences:

Scrambled: 5′-CGUACGCGGAAUACUUCGAUU-3′
MCRS1: 5′-GGCAUGAGCUCUCCGGAC-3′

Cells treated twice with siRNA as described above were collected after 72 h for either Western blotting or imaging (i.e., immunofluorescence microscopy or electron tomography).

#### Gel electrophoresis and Western blots.

Protein lysates from HeLa cells were prepared by resuspending cell pellets in RIPA buffer containing 50 mM Tris-HCl, pH 7.4, 150 mM NaCl, 2 mM ethylene glycol-bis(β-aminoethyl ether)-*N*,*N*,*N*′,*N*′-tetraacetic acid (EGTA), 1% Triton X-100 (Sigma-Aldrich, St. Louis, MO), 0.5% deoxycholic acid (DOC) (Sigma-Aldrich, St. Louis, MO). and 0.1% SDS supplemented with protease inhibitors (Sigma-Aldrich, St. Louis, MO). After incubation on ice for 15 min, the lysates were centrifuged at 4°C at maximum speed for 15 min. The protein content of the lysates was determined by using the Pierce BCA kit (Thermo Fisher Scientific) and following the manufacturer’s instructions. For each run, 50 μg of protein lysate was diluted in loading buffer 5×, boiled for 10 min, and loaded in a 10% SDS–PAGE gel. Gels were run at 120 V for 90 min.

For Western blots, a semidry transfer was done to blot the proteins in a 0.45 μm nitrocellulose membrane (GE Healthcare, Chicago, IL). Proteins were transferred for 90 min at 65 mA. The membrane was blocked using 5% milk (Sigma-Aldrich, St. Louis, MO) in Tris-buffered saline (TBS) at 4°C overnight. Primary antibodies (MCRS1, home-made; tubulin [DM1A], T6199, Sigma-Aldrich, St. Louis, MO) were incubated in 0.1% Tween-20 (Sigma-Aldrich, St. Louis, MO) in TBS for 1 h at room temperature. Secondary antibodies conjugated with AlexaFluors (AlexaFluors800—MCRS1; AlexaFluors680—α-tubulin) were incubated in the same buffer at room temperature for 45 min. Blots were developed using an Odyssey Infrarred Imaging System (Li-cor, Lincoln, NE).

### Light microscopy

For immunofluorescence, HeLa cells were grown on 18-mm round coverslips (Marienfeld, Germany) in six-well plates. Cells were fixed by immersion in cold methanol (–20°C) for 10 min. Cells were then blocked and permeabilized in 0.5% bovine serum albumin (BSA) (Panreac, Barcelona, Spain) and 0.1% Triton X-100 (Sigma-Aldrich, St. Louis, MO) in 1× phosphate-buffered saline (PBS) for 30 min at room temperature. Primary antibodies (MCRS1, homemade; tubulin [DM1A], T6199, Sigma-Aldrich, St. Louis, MO) were incubated in the same blocking buffer for 1 h at room temperature. Secondary antibodies conjugated with AlexaFluors 488 and Hoechst to stain DNA (Life Technologies, Carlsbad, CA) were diluted in blocking buffer 1:1000 and incubated at room temperature for 45 min. Coverslips were mounted. in 9.6% (wt/vol) Mowiol, containing 24% (wt/vol) glycerol (Sigma-Aldrich, St. Louis, MO) and 0.1 M Tris-Cl.

After immunostaining, samples were visualized by light microscopy to select MCRS1-depleted cells. The samples were visualized with a LEICA SP5 microscope (Leica Microsystems, Austria) equipped with a water 63× objective lens (Leica HCX PL APO 63×/1.2 W) and a PMT Leica hybrid detector camera (HyD) (Dresden). Images were acquired using a 488 nm argon laser with a HyD sensor set to collect light from 500 to 550 nm and a 594 nm diode laser with a sensor set from 620 to 730 nm. Chromosome alignment at the metaphase plate was considered as an indicator of the mitotic stage. Selected mitotic cells in the metaphase stage were further characterized by measuring both the half-spindle and the MT angle using Fiji (Schindelin *et al.*, 2012) (Figure 2B). For this, the maximum-intensity projection tool was used for the z-stacks corresponding to the α-tubulin staining in the selected spindles. A threshold was set for the projected images to create a binary mask. The angles were measured manually using the angle tool. The half-spindle angle was measured to analyze the shape of the spindle poles, and it was measured by calculating the angle between the metaphase plate and the spindle pole (Figure 2B). The MT angle was measured to analyze the degree of bending of the outer MTs. We measured the outline of the quarter-spindle by calculating the angle between the metaphase plate and the spindle pole (Figure 2B). Differences in the measured angles in both siScramble and siMCRS1 spindles were assessed by applying a linear regression model.

### Electron microscopy

#### Sample preparation.

In preparation for electron microscopy, 3- and 6-mm sapphire disks (M. Wohlwend GmbH, Switzerland) were prepared for the attachment of cells. The mitotic fraction of the cell culture was then collected by applying the “shake-off” technique (Kiewisz *et al*., 2021). Briefly, flasks with HeLa cells were subjected to two “shake-off” rounds. Flasks were hit against the surface of the bench to detach mitotic cells. The collected cells were then centrifuged at 1200 rpm at 37°C for 4 min, and the cell pellets were resuspended in 1 ml of DMEM (Thermo Fisher Scientific, Waltham, MA) supplemented with 10% BSA (Thermo Fisher Scientific, Waltham, MA) and 10%fetal bovine serum (Thermo Fisher Scientific, Waltham, MA). Next, the cells were allowed to attach to carefully cleaned sapphire disks for 10 min at 37°C. Cleaning of the sapphire disks included immersion in a Piranha solution (1:1 H_2_SO_4_ and H_2_O_2_, vol/vol), coating with poly-l-lysine 0.1% (wt/vol), and drying at 60°C for 2 h. Finally, the disks were incubated with a 1:10 solution of fibronectin (Sigma-Aldrich, St. Louis, MO) in PBS at 37°C for 2 h before use.

#### High-pressure freezing and freeze substitution.

Cells attached to 3-mm sapphire disks were cryoimmobilized using an EM ICE (Leica Microsystems, Austria), and 6-mm sapphire disks were frozen by using a Compact 03 high-pressure freezer (M. Wohlwend GmbH, Switzerland). For each freezing round with the EM ICE, a type-A aluminum carrier (Leica Microsystems, Austria) with the 100-µm indentation facing up was placed in the specimen loading device of the freezer. The cavity of the carrier was then filled with 5 µl of DMEM containing 10% BSA, and the sapphire disk with the attached HeLa cells facing down was placed onto the carrier. Next, a spacer ring was mounted on top of the sample and the freezing started immediately*.* For using the Compact 03 high-pressure freezer, the 6-mm sapphire disks with the mitotic cells facing down were placed on a type-A aluminum planchette with the 40-µm-deep cavity prefilled with warm DMEM supplemented with 10% BSA. Closed carriers were then placed in the specimen holder, clamped with the holder arm, and immediately cryoimmobilized. This approach as developed for freezing with the Compact 03 allowed for a quick inspection of the assembled specimens, so that only samples without trapped air were further processed for high-pressure freezing (Kiewisz *et al.*, 2022). Using both freezers, samples were frozen at ∼2000 bar with a cooling rate of ∼20,000°C/s (Reipert *et al.,* 2004). After freezing, samples were stored in liquid nitrogen until further use. Freeze substitution was carried out as previously described (Muller-Reichert *et al.*, 2003). Briefly, samples were transferred to cryo-vials filled with anhydrous acetone containing 1% osmium tetroxide (EMS, USA) and 0.1% uranyl acetate (Polysciences, USA). Freeze substitution was done using an automatic freeze substitution machine (EM AFS, Leica Microsystems, Germany). Samples were kept at –90°C for 1 h and then warmed up to –30°C in steps of 5°C/h and maintained at –30°C for 5 h. Next, the temperature was increased to 0°C in steps of 5°C/h. Finally, after reaching 0°C, samples were washed three times with pure anhydrous acetone at room temperature.

#### Sample embedding, preselection of mitotic cells and ultramicrotomy.

For resin embedding, the samples were placed in flow-through chambers (Leica, Germany) and infiltrated with Epon/Araldite (EMS, Hatfield, PA) in three steps of 1 h each with increasing concentration of resin: 1:3, 1:1, and 3:1 (resin:acetone, wt/vol), followed by a single step overnight in pure resin at room temperature (Müller-Reichert *et al.*, 2003). Then, samples were polymerized at 60°C for 48 h. After polymerization the plastic samples were removed from the flow-through chambers. Using a razor blade, the sapphire disks were then removed from the resin blocks to expose the embedded cells for further processing as described previously (Kiewisz *et al.*, 2022). This procedure was chosen to avoid any remounting of thin layers of resin on dummy blocks.

To screen for cells in metaphase, the resin blocks were observed from the top using an upright brightfield microscope (Zeiss, Germany). The two main features used to select cells in metaphase were a rounded shape and a distinguishable metaphase plate (Kiewisz *et al.*, 2021). Serial semithick (300 nm) sections were cut using an EM UC6 ultramicrotome (Leica Microsystems, Austria) and collected on Formvar-coated slot grids. Samples were poststained with 2% uranyl acetate for 10 min followed by 0.4% Reynold’s lead citrate solution (Science Services, Germany). Colloidal 15-nm gold particles (British Biocell International, UK) were attached to both sides of the sections mounted on the grids to serve as fiducial markers for tomographic reconstruction.

#### Final staging of preselected spindles.

The serial sections were imaged using a TECNAI T12 Biotwin transmission electron microscope (Thermo Fisher Scientific, Waltham, MA) operated at 120 kV and equipped with an F214 charge-coupled device (CCD) camera (TVIPS GmbH, Germany). Images of whole cells in metaphase were acquired at 1200× magnification using EMMenu Software (TVIPS GmbH, Germany). To choose a cell for electron tomography, the metaphase plate had to be correctly formed when looking at the chromosome area in 3D and for each chosen cell the half-spindle and the MT angle were measured in the 3D volumes as described (see the *Light microscopy* section). For this, the EM stacks were projected in 3D. The chromosome and microtubule area were estimated by manually labeling the chromosome and MT area. Both the half-spindle angle and MT angle were finally calculated using the ZIB Amira software (Zuse Institute Berlin, Germany).

### Three-dimensional reconstruction by electron tomography

#### Data acquisition and calculation of tomograms.

Electron tomography was performed on the selected metaphase cells as previously described (Kiewisz *et al.*, 2021). Briefly, a series of tilted views were recorded using a TECNAI F30 transmission electron microscope (Thermo Fisher Scientific, Waltham, MA) operated at 300 kV and equipped with a Gatan US1000 2K × 2K CCD camera. The SerialEM software package was used for the acquisition and montaging of data sets (Mastronarde, 2003, 2005). For dual-axis electron tomography, images were captured every 1.0° over a ±60° range at a pixel size of 2.32 nm. For a recording of the second axis, grids were rotated by 90° and another series of tilted views was acquired (Mastronarde, 1997). Tomograms were calculated by using the IMOD software package (Mastronarde and Held, 2017). To increase the sample number, we acquired volumes of quarters of the cells (see Supplemental Table S1).

#### Segmentation of MTs and stitching of serial tomograms.

MTs were semiautomatically segmented using the ZIB Amira (Zuse Institute Berlin, Germany) software package (Lindow *et al.*, 2021) as previously described (Weber *et al.*, 2012; Redemann *et al.*, 2014; Lindow *et al.*, 2021). After manual correction of MT segmentation, the serial tomograms of each recorded cell were stitched using the segmented MTs as alignment markers (Weber *et al.*, 2014; Lindow *et al.*, 2021).

#### Z-correction of stacked tomograms.

Each stack of serial tomograms was expanded in *Z* to correct for a sample collapse during the data acquisition (McEwen and Marko, 1999). We corrected this shrinkage by applying a Z-factor to the stacked tomograms (O’Toole *et al.*, 2020; Kiewisz *et al.*, 2021, 2022). Taking the microtome setting of around 300 nm, we multiplied this value by the number of serial sections. For each spindle, we also determined the thickness of each serial tomogram and then calculated the total thickness of the reconstruction. The *Z*-factor was then determined by dividing the actual thickness of each stack of tomograms by the total thickness as determined by the microtome setting. Such calculated Z-factors were then applied to our full spindle reconstructions.

### Quantitative analysis of tomographic data

For quantitative analysis of the tomographic data, the automatic spatial graph analysis (ASGA) software tool (Kiewisz and Müller-Reichert, 2021) was used to measure the outer-kinetochore distance, the number of MTs per k-fiber, and the distribution and morphology of the MT minus ends. Quantitative analyses were carried out essentially as described (Kiewisz *et al.*, 2022).

#### Outer-kinetochore distance.

The outer-kinetochore distance was used as a readout of the mitotic stage of the selected cells (Maresca and Salmon, 2009). To measure the outer-kinetochore distance, the neighboring sister kinetochores were identified in the 3D models. The center of each kinetochore was defined as the median position of all KMT plus ends associated with each selected outer kinetochore, and the outer-kinetochore distance was then calculated as the 3D Euclidean distance between the defined median centers of each kinetochore pair. The outer-kinetochore distances were determined for control, siScramble, and siMCRS1 cells, and values were compared using a linear regression model.

#### Number of KMTs per k-fiber.

MTs associated with the kinetochores were defined as KMTs. Accordingly, the average number of KMTs was determined for each experimental condition. Analysis of the number of KMTs per k-fiber was done using a GLM in R, applying a Poisson likelihood and a log link function. The Poisson distribution is generally used to model discrete positive outcomes such as the number of MTs when the numbers are small and cannot be approximated by a normal distribution (*n* < 20).

#### Distribution of MT minus ends.

The polarity of KMTs was assigned as follows. The end of each KMT associated with a kinetochore was assumed to be the plus end and the other end the minus end. To analyze the position of the KMT minus ends in the metaphase spindles, the 3D Euclidean distance of each KMT end to both spindle poles (i.e., to the center of the mother centriole of the respective pole) was determined. Then, the KMT minus end was determined as the end closest to one of the spindle poles. In addition, the relative position of KMT minus ends along the pole-to-pole axis was calculated. The relative position of each minus end is given as the normalized position between the kinetochore (position = 1) and the mother centriole (position = 0) along the pole-to-pole axis. The distribution of relative positions of KMT minus ends (mean, ±SD) is as an average density distribution for each condition. For non-KMTs, the end closer to the nearest centriole pair was defined as the minus end. The relative distance for the non-KMTs was calculated between the average kinetochore (position = 1) and the mother centriole (position = 0).

#### Tortuosity of KMT.

To analyze the global tortuosity of the KMTs, the ratio of the spline length and the 3D distance between the plus and the minus ends of each KMT was measured (Kiewisz *et al.*, 2022). The correlation of KMT tortuosity and their length is shown by a fitted curved line calculated with a logarithmic function.

#### Morphology of MT ends.

MT ends were annotated by manual segmentation. MT ends were manually classified as open, closed, or undefined by two different observers using the end classifier in the ZIB Amira software package (Stalling *et al.*, 2005). To ensure an unbiased manual annotation of the end morphology, a random 3D view of each MT was presented to the analyst without knowledge of the MT identity (either KMT or non-KMT). The polarity of the MT ends (either plus or minus) and location (relative distance) within the spindle were determined after the classification of the end morphology.

Analysis of the MT end morphology was modeled as a binary outcome (open vs. closed), such that the number of open ends was naturally drawn from a binomial distribution depending on the true unobserved proportion of open ends. To model the dependency of the proportion of open ends on the distance to the spindle pole, we discretized the data into 16 different intervals and counted the open ends called by each observer. We used a third-order b-spline on the underlying logit transformation of the proportion of open ends to jointly infer the proportion of open ends at each possible distance from the pole. We then added the observer as a random effect to consider the differences in the classification made by the two independent observers.

#### Error analysis of tomographic data.

Errors in automatic MT segmentation and in the stitching of serial tomograms have been discussed previously (Redemann *et al.*, 2014; Kiewisz *et al.*, 2021, 2022; Lindow *et al.*, 2021). As for the segmentation of MTs, the error associated for our approach is in the range of 5–10% (Weber *et al.*, 2012). Each individual MT in our reconstruction was checked manually for the correct tracing of both ends.

In previous publications (Weber *et al.*, 2014; Redemann *et al.*, 2017; Lindow *et al.*, 2021), we estimated the overall quality of the MT stitching by analyzing the distribution of MT endpoints in the Z-direction (i.e., normal to the plane of the slice). We expect to find approximately the same density of MT endpoints along the Z-direction of each serial-section tomogram. Therefore, if the density of endpoints after matching is approximately the same along the Z-direction of the serial-section tomograms, we can assume that the number of artificial points that have been introduced at the interfaces of the serial sections is negligible (Kiewisz *et al.*, 2021, 2022).

### Data availability

Tomographic data have been uploaded to the TU Dresden Open Access Repository and Archive system (OpARA) and are available as open access: https://opara.zih.tu-dresden.de/xmlui/handle/123456789/5750.

The code used to perform quantitative analysis of MT organization in spindles has been uploaded to the GitHub repository and is available as open access under the GPL v3.0 license: https://github.com/RRobert92/ASGA_3DViewer.

Data and code for statistical analysis of MT ends can be accessed at https://bitbucket.org/cmartiga/kfibers/src/master/.

## Abbreviations used:

aK: average position for all kinetochores
AMTs: astral microtubules
ASGA: automatic spatial graph analysis
BSA: bovine serum albumin
CCD: charge-coupled device camera
CI: credible interval
Ctrl: control
3D: three-dimensional
DMEM: Dulbecco’s Modified Eagle Medium
DOC: deoxycholic acid
EGTA: ethylene glycol-bis(β-aminoethyl ether)- *N*, *N*, *N*′, *N*′-tetraacetic acid
EM: electron microscopy
ET: electron tomography
GLM: generalized linear model
γ-TuRC: gamma-tubulin ring complex
IMTs: interpolar microtubules
K-fibers: kinetochore fibers
KMTs: kinetochore microtubules
MAPs: microtubule-associated proteins
MCRS1: microspherule protein 1
MTs: microtubules
non-KMTs: non-kinetochore microtubules
PBS: phosphate-buffered saline
siRNA: small interferring RNA
TBS: Tris-buffered saline
U2OS: human bone osteosarcoma epithelial cells.
