# Supplementary material for: MCRS1 modulates the heterogeneity of microtubule minus-end morphologies in mitotic spindles
Source: Mol Biol Cell. 2022 Dec 15;34(1):ar1. doi: 10.1091/mbc.E22-08-0306-T (PMC9816640; doi:10.1091/mbc.E22-08-0306-T)
Supplement: Supplementary file 8 [file mbc-34-ar1-s001.pdf]

Supplementary Materials

Molecular Biology of the Cell

Laguillo-Diego *et al*.

SUPPLEMENTAL MATERIALS

Molecular Biology of the Cell

Laguillo-Diego A., Kiewisz R. et al.

SUPPLEMENTAL MOVIE LEGENDS

Video 1. Generation of a 3D model assembled by joining serial electron tomograms
Series of tomograms and corresponding 3D model of a control spindle #3. The video illustrates the stacking of serial tomograms to increase the volume of reconstruction. KMTs are shown as red lines. This video corresponds to the spindle shown in **Fig. 1C**.

Video 2. Three-dimensional model of a low-resolution spindle as used for the analysis of spindle geometry in a siScramble cell
Series of electron micrographs of a spindle in a siScramble cell #2. The stacking of serial tomograms to generate a 3D model of the spindle volume is illustrated. The spindle on each section is indicated by a transparent gray area. Chromosomes are shown in blue. This video corresponds to the spindle shown in **Fig. 2D, left panel**.

Video 3. Three-dimensional model of a low-resolution spindle as used for the analysis of spindle geometry in a siMCRS1 cell
Series of electron micrographs of a spindle in a siMCRS1 cell #5. The stacking of serial tomograms to generate a 3D model of the spindle volume is illustrated. The spindle on each section is indicated by a transparent gray area. Chromosomes are shown in blue. This video corresponds to the spindle shown in **Fig. 2D, right panel**.

Video 4. Generation of a 3D model from joined serial electron tomograms displaying spindle siMCRS1 #1
Series of tomograms and corresponding 3D model of spindle siMCRS1 #1. The stacking of serial tomograms to increase the tomographic volume is illustrated. Non-KMTs are shown as yellow lines, and KMTs are illustrated in red. This spindle reconstruction is not shown in any of the presented figures but has been added to show that the morphological alterations upon MCRS1-silencing are consistent between different 3D models. This video corresponds to spindle siMCRS1 #1 as given in **Table 1**.

Video 5. Generation of a 3D model from joined serial electron tomograms displaying spindle siMCRS1 #5
Series of tomograms and corresponding 3D model of spindle siMCRS1 #5. The stacking of serial tomograms to increase the tomographic volume is illustrated. KMTs are shown as red lines. This video corresponds to the spindle shown in **Fig. 3A**.

Video 6. KMTs end morphology in spindle siScramble #2
Three-dimensional model of KMTs with annotated end morphology. KMTs are indicated as black lines. Open KMT ends are labeled with green spheres, closed ends with purple spheres and undefined ends with white spheres. This video corresponds to the spindle shown in **Fig. 3A**

Video 7: KMT end morphology in spindle siMCRS1 #5
Three-dimensional model of KMTs with annotated end morphology. KMTs are indicated as black lines. Open KMT ends are labeled with green spheres, closed ends with purple spheres and undefined ends with white spheres. This video corresponds to the spindle shown in **Fig 3A.** and **Fig. 4F**

SUPPLEMENTAL TABLES

Supplemental Table 1. Tomographic data sets as used throughout this study

Metadata/ data set	Original data set	Spindle reconstruction	No. of serial sections[1]/ Z-factor	Estimated tomographic volume (μm^3)	Data size (Gb)
Ctrl #1	T_0475	Full (divided into quarters)	22 / 1.30	144	44.3
Ctrl #2	T_0479	Full (divided into quarters)	29 / 1.40	263	74.2
Ctrl #3	T_0550_wt_1	Quarter	17 / 1.30	114	30
siScr #1	T_0506	Quarter	12 / 1.25	58	22.4
siScr #2	T_0550_scr_3	Quarter	14 / 1.30	78	28.3
siMCRS1 #1	T_0506_mcrs1_2	Quarter	18 / 1.32	94	26.8
siMCRS1 #2	T_0506_mcrs1_4	Quarter	18 / 1.35	87	24.9
siMCRS1 #3	T_0506_mcrs1_5	Quarter	17 / 1.30	79	22.8
siMCRS1 #4	T_0550_mcrs1_2	Quarter	16 / 1.35	112	32.1
siMCRS1 #5	T_0550_mcrs1_3	Quarter	21 / 1.36	110	31.6

[1] Average section thickness is 300 nm.

Supplemental Table 2. Quantitative analysis of the tomographic reconstructions as used throughout this study

Data set	No. of MTs	No. of KMTs	No. of KMTs in the reconstructed volume	No. of k-fibers	Pole-to-pole distance (µm)	Distance between outer kinetochores (µm)
Ctrl #1 (Kiewisz et al., 2022)	4884	797	748	92	7.16	1.08 ±0.20 (n=43)
Ctrl #2 (Kiewisz et al., 2022)	8047	1102	1072	110	10.39	1.24 ±0.21 (n=50)
Ctrl #3	-	407	205	22	-	1.15 ±0.22 (n=22)
siScr #1	2913	397	203	20	-	1.02 ±0.20 (n=17)
siScr #2	6221	344	205	24	-	1.06 ±0.21 (n=28)
siMCRS1 #1	2884	224	50	8	-	1.10 ±0.24 (n=20)
siMCRS1 #2	2451	169	57	10	-	1.12±0.23 (n=10)
siMCRS1 #3	3635	301	159	18	-	1.06 ±0.11 (n=6)
siMCRS1 #4	5624	348	180	29	-	1.02 ±0.18 (n=28)
siMCRS1 #5	3811	484	273	39	-	1.13±0.20 (n=35)

Supplemental Table 3. Quantitative analysis of MT minus-end morphologies

End morphology	KMT minus ends				KMT plus ends			
	Total no.	Matching open ends	Matching closed ends	Undefined ends	Total no.	Matching open ends	Matching closed ends	Undefined ends
**Control** (Ctrl #1, #2, #3)	2239	682	385	532	1841	1509	6	218
**siScramble** (siScr #1, #2)	407	109	66	137	432	350	2	47
**siMCRS1** (siMCRS1 #1, #2, #3, #4, #5)	726	297	90	185	847	692	2	108
**End morphology**	**Non-KMT minus ends**							
	Total no. of ends	Matching open ends	Matching closed ends	Undefined ends				
**Control** (Ctrl #1, #2, #3)	1511	521	209	427				
**siScramble** (siScr #1, #2)	1155	278	59	591				
**siMCRS1** (siMCRS1 #1, #2, #3, #4, #5)	6654	1876	509	3539				

SUPPLEMENTAL FIGURES

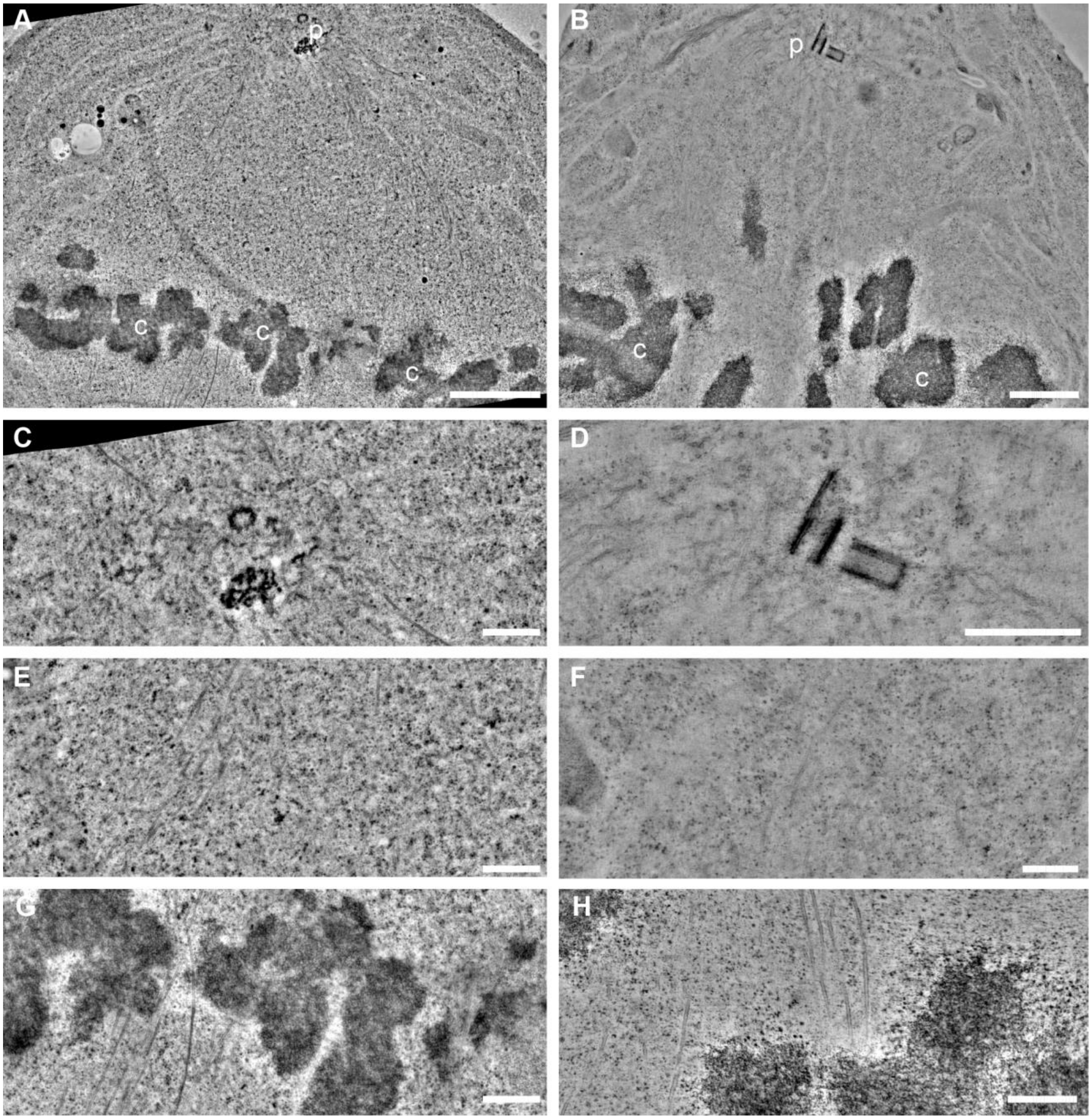

SUPPLEMENTAL FIGURE S1. Illustration of metaphase in control and siMCRS1 cells.

Tomographic slices are shown to illustrate the morphology of control (Ctrl #3, left column) and siMCRS1 cells (#5, right column). (**A–B**) Low-magnification images showing regions between a spindle pole (p) to chromosome (c) region. Scale bar, 1 µm. (**C–D**) Centrioles and MTs at higher magnification. Scale bars, 500 nm. (**E–F**) Regions between the spindle poles and the chromosomes. Scale bars, 500 nm. (**G–H**) Kinetochores with associated KMTs. Scale bars, 500 nm.

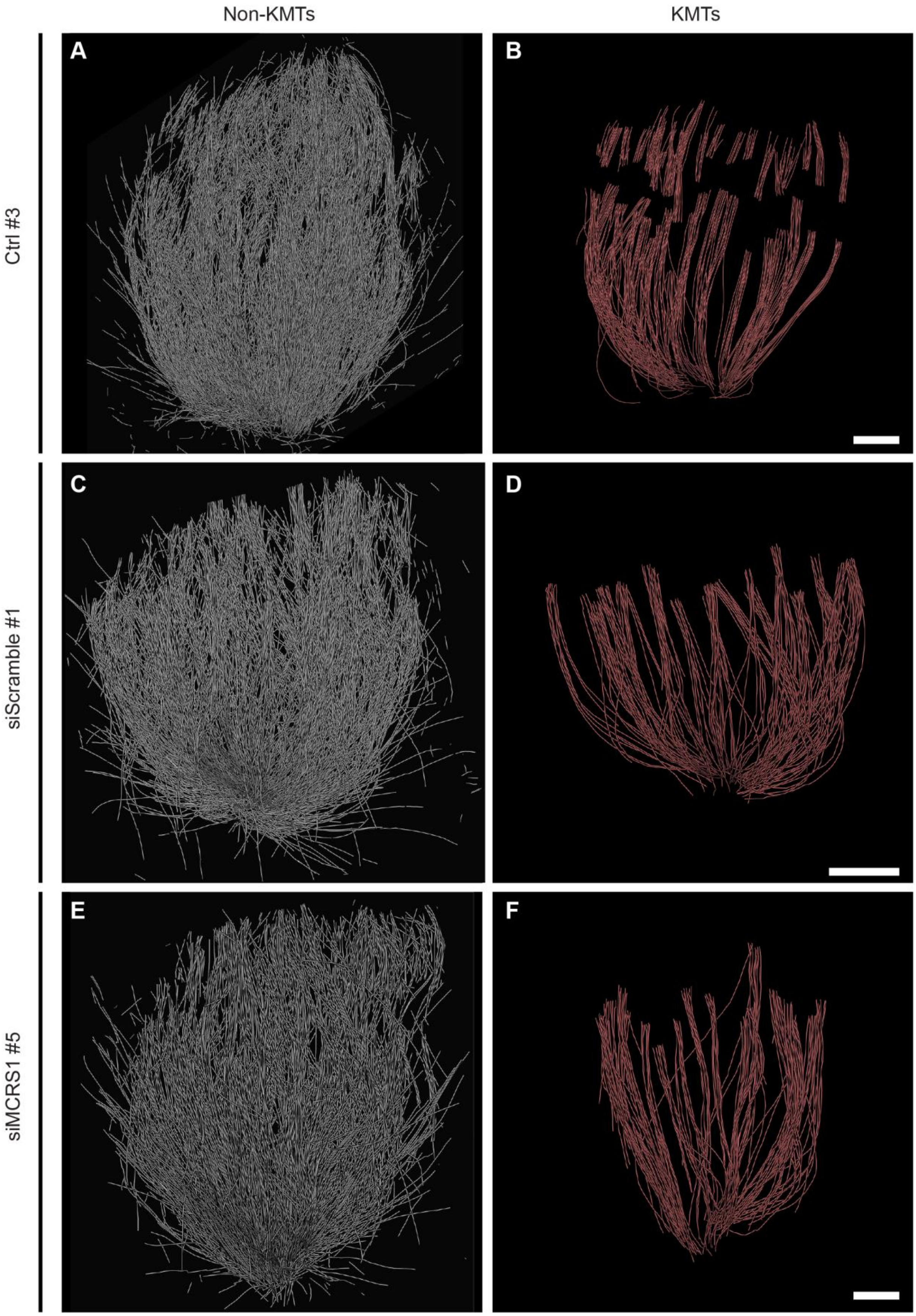

Non-KMTs
KMTs
Ctrl #3
siScramble #1
siMCRS1 #5
A
B
C
D
E
F

SUPPLEMENTAL FIGURE 2. Three-dimensional modes of metaphase spindles as observed by large-scale electron tomography.

(**A**) Three-dimensional model of non-KMTs in a control spindle (#3). (**B**) Illustration of only KMTs in a control spindle (#3) as shown in B. (**C**) Model of non-KMTs in a Scrambled spindle (#1). (**D**) Illustration of only KMTs in the Scrambled spindle (#1) as shown in C. (**E**) The model of non-KMTs in a siMCRS1 spindle (#5). (**F**) Illustration of only KMTs in the siMCRS1 spindle (#5) as shown in D. Scale bars, 1 μm.

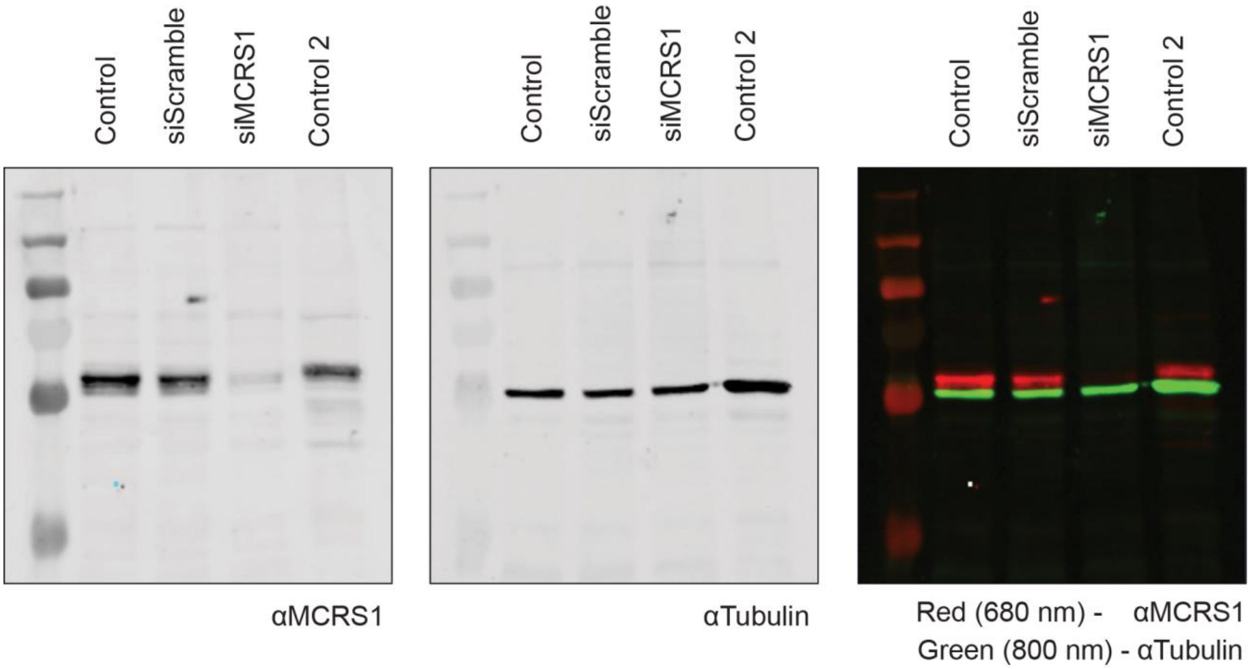

SUPPLEMENTAL FIGURE 3. Analysis of protein levels by Western Blot.

Full western blots corresponding to the cropped Western Blot shown in **Fig 1A**. Lysates from two controls, siScramble and siMCRS1 cells were run on SDS-PAGE. Left, the membrane was probed with the anti-MCRS1 antibody and re-probed with an anti-αTubulin antibody as loading control (middle). The overlay of both signals is shown on the right (anti-MCRS1, red; anti-Tubulin, green).

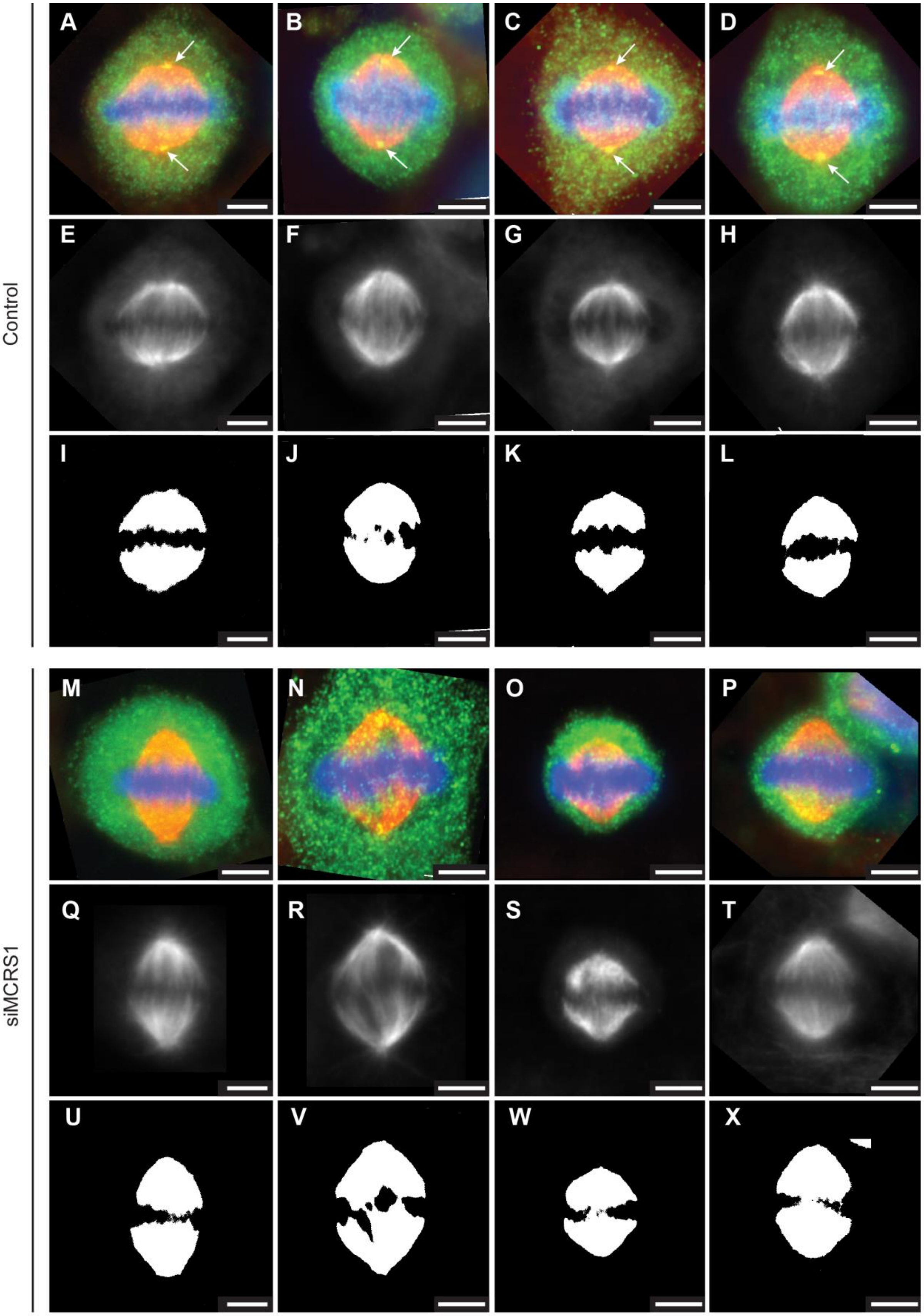
Control
siMCRS1
A
B
C
D
E
F
G
H
I
J
K
L
M
N
O
P
Q
R
S
T
U
V
W
X

SUPPLEMENTAL FIGURE 4. Angle analysis for control and siMCRS1 depleted HeLa cells

Set of fluorescent images showing the steps analysis. (**A-D**, and **M-P**) Raw images. MCRS1 is shown in green, DNA in blue and tubulin in magenta. MCRS1 staining can be observed as tiny spots at spindle poles in the control cell (arrows) (**E-H**, and **Q-T**) The middle row shows extracted tubulin channel after z-projection. (**I-L**, and **U-X**) Automatic threshold image. A created binary mask is used to define spindle and MT angles. Scale bars, 5 μm.

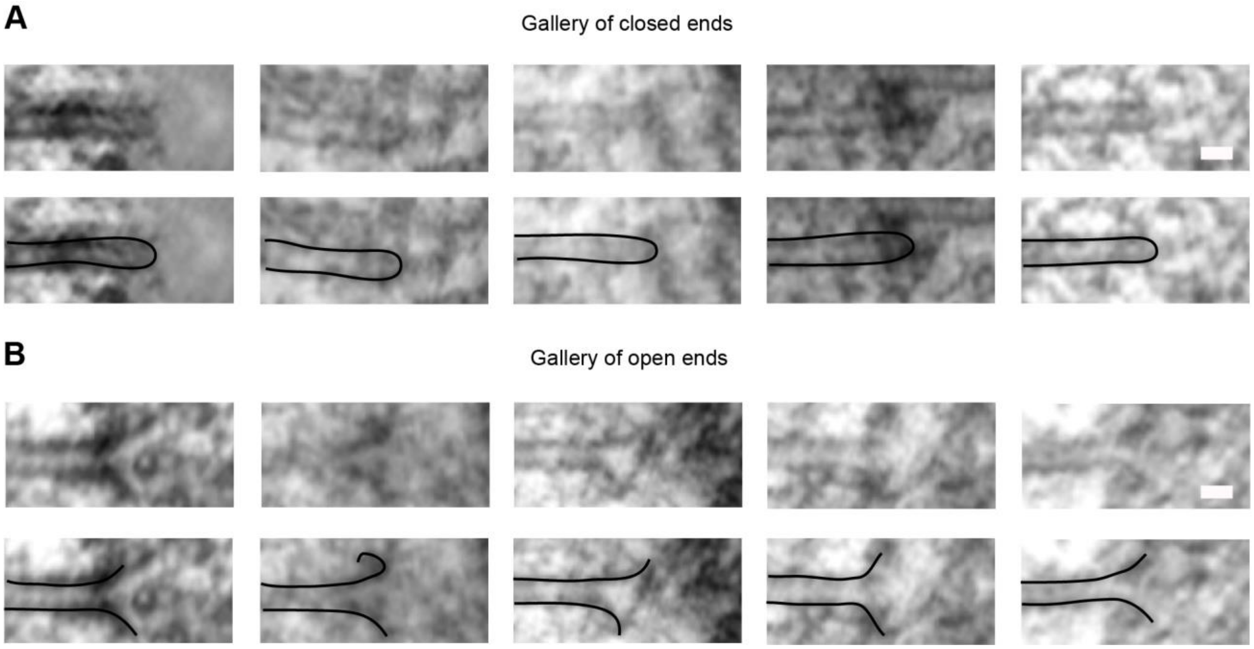

SUPPLEMENTAL FIGURE 5. Gallery of MT ends morphology as observed by electron tomography

(**A**) Representative collection of images showing closed MT ends (top row, tomographic slices; lower row, tomographic slices with overlays). The polarity of the MTs is not assigned. Scale bar, 25 μm. (**B**) Collection of images showing open MT ends. Scale bars, 25 μm.

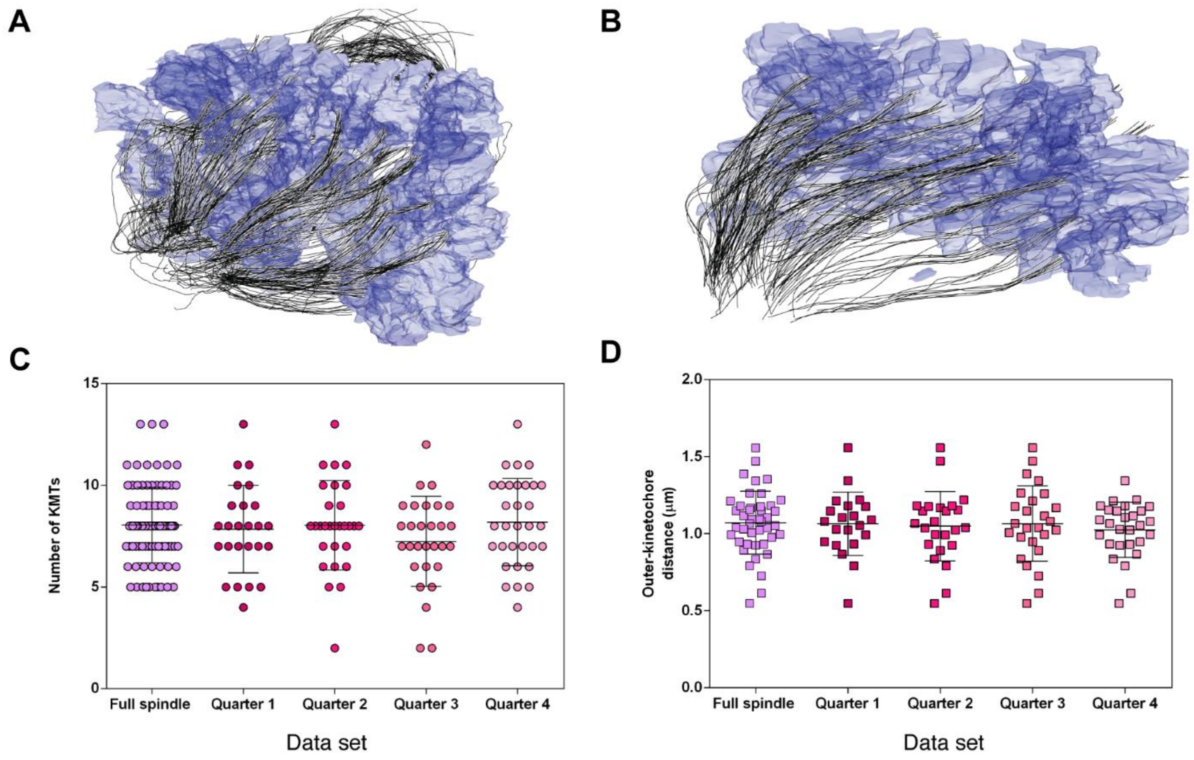

SUPPLEMENTAL FIGURE 6. Comparison of the characteristics of a full spindle *versus* their quarters

(**A**) Perspective view of a full 3D model showing the KMTs in a control spindle #1. KMTs are shown as black lines, with chromosomes in gray. (**B**) Perspective view of a 3D model showing the KMTs in a quarter spindle #1. (**C**) Scatterplots showing the number of KMTs per k-fiber for a full spindle #1 and the four-quarters of the same spindle. The midline shows the mean, and the error bars represent ±SD. The mean numbers of KMTs for the full spindle is 8.04 ±1.86 µm (mean ±SD, n=93), and for the quarter (#1: 7.84 ±2.15 µm, n=25; #2: 8.03 ±2.20 µm, n=29; #3: 7.24 ±2.21 µm, n=29; and #4: 8.19 ±2.15 µm, n=32). There are no significant differences among the mean values for the full spindle and the quarter spindles after ANOVA analysis (p-value = 0.3791). (**D**) Scatterplot of the outer-kinetochore distance in the full spindle *versus* the spindle quarters. The midline shows the mean, and the error bars represent ±SD. The mean outer-kinetochore distances for the full spindle is 1.07 ±0.20 µm (n=43), and for the quarter (#1: 1.06 ±0.20 µm, n=21; #2: 1.05 ±0.23 µm, n=24; #3: 1.06 ±0.24 µm, n=26; and #4: 1.03 ±0.18 µm, n = 28). There are no significant differences among the mean values for the full spindle and the quarter spindles after ANOVA analysis (p-value = 0.9192).

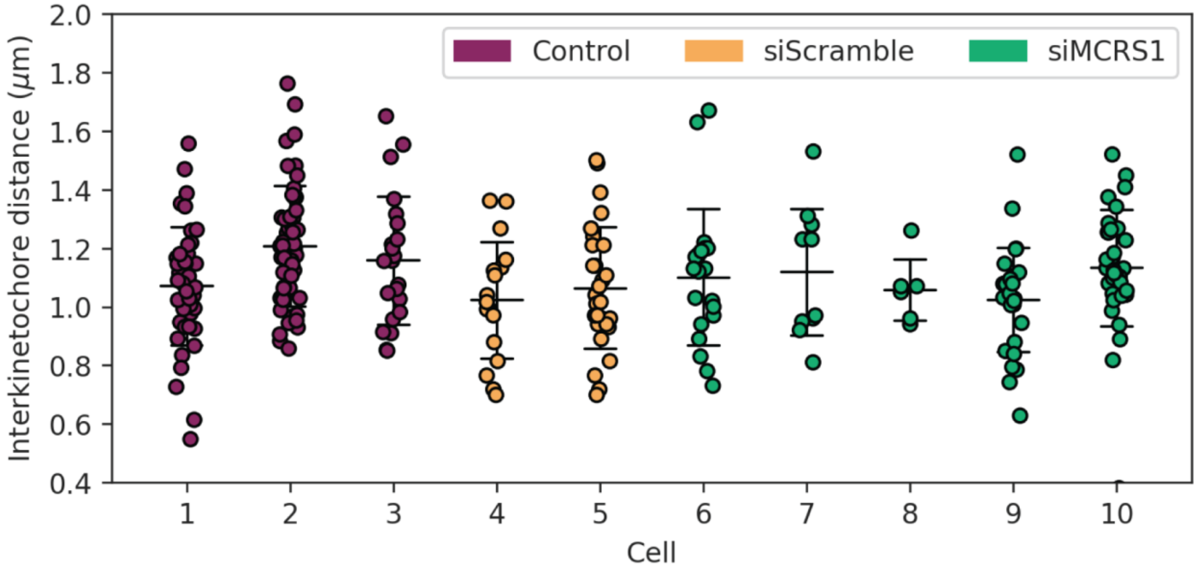

SUPPLEMENTAL FIGURE 7. Comparative analysis of outer-kinetochore distances in 3D models of control, siScramble and siMCRS1-depleted cells.

Scatter plots of the inter-kinetochore distances as measured for each of the 3D-reconstructed spindles. The mean inter-kinetochore distance is 1.14 μm (n=114) in control cells, 1.05 μm (n=44) in siScramble cells and 0.99 μm (n=98) in siMCRS1 cells. A linear regression model did not reveal significant differences between siScramble versus Control p-value = 0.108 and siScramble versus siMCRS1 (p-value=0.240). The numbers for each cell are matching the data sets as given in Table 1. Cells #1-2 correspond to full spindle reconstructions and cells #3-10 to quarter spindles.

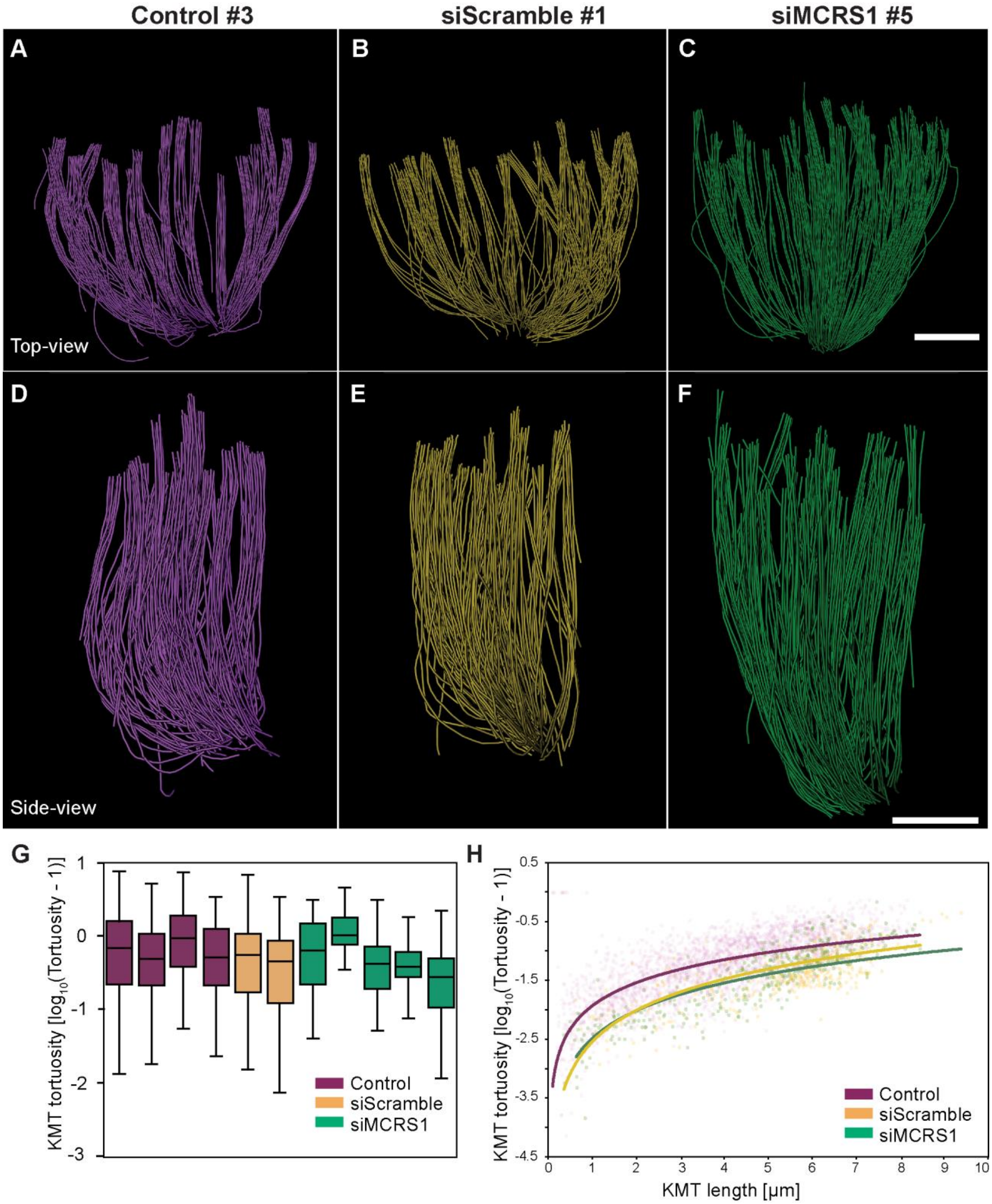
Control #3
siScramble #1
siMCRS1 #5
A
B
C
Top-view
D
E
F
Side-view
G
KMT tortuosity [log10(Tortuosity - 1)]
1
0
-1
-2
-3
Control
siScramble
siMCRS1
H
KMT tortuosity [log10(Tortuosity - 1)]
0.5
-0.5
-1.5
-2.5
-3.5
-4.5
0
1
2
3
4
5
6
7
8
9
10
KMT length [µm]
Control
siScramble
siMCRS1

SUPPLEMENTAL FIGURE 8. Analysis of KMT tortuosity in a 3D model of control, siScramble and siMCRS1 cells

(**A-C**) Orthogonal projections (top views) of KMTs in representative 3D models of ctrl (#3, left panel, lines in magenta), siScramble (#2, mid panel, lines in yellow) and siMCRS1 (#5, right panel, lines in green). Scale bar, 1 µm. (**D-F**) Side-views of the orthogonal projections of KMTs as shown in A-C. Scale bars, 1 µm. (**G**) Boxplot showing the logarithmic representation of KMT tortuosity as measured for each of the spindles. The boxes show the upper and lower quartiles, the whiskers show the minimum and maximal values excluding outliers; the line inside the box indicates the median (Control: n=3164, siScramble: n=408, siMCRS1: n=727). (**H**) Lineplot showing a logarithmic representation of KMT tortuosity against the KMT length for each data point. The lines show a logarithmic fit for each condition.
